# *Cutibacterium acnes* strains associated with bone prosthesis infections cannot evade the host immune system

**DOI:** 10.3389/fimmu.2024.1468709

**Published:** 2024-11-27

**Authors:** Léa Thoraval, Min Tang-Fichaux, Christine Guillaume, Jennifer Varin-Simon, Claire Dumortier, Johan Sergheraert, Fabien Lamret, Mélanie Bonhomme, Frédéric Laurent, Jérôme Josse, Sophie C. Gangloff, Céline Mongaret, Fany Reffuveille, Frédéric Velard

**Affiliations:** ^1^ Université de Reims Champagne-Ardenne, BIOS, Reims, France; ^2^ Université de Reims Champagne-Ardenne, CHU Reims, BIOS, Pôle de Médecine Bucco-Dentaire, UFR Odontologie, Reims, France; ^3^ Centre International de Recherche en Infectiologie (CIRI), Université de Lyon, Inserm, U1111, Université Claude Bernard Lyon 1, CNRS, UMR5308, ENS de Lyon, Lyon, France; ^4^ Université de Reims Champagne-Ardenne, BIOS, UFR Pharmacie, Reims, France; ^5^ Université de Reims Champagne-Ardenne, CHU Reims, BIOS, Service Pharmacie, Reims, France

**Keywords:** inflammation, *Cutibacterium acnes*, biofilm, bone and joint infection, human primary neutrophils

## Abstract

**Introduction:**

*Cutibacterium acnes* is a commensal skin bacterium that is involved in bone prosthesis infections (BPIs) and presents low-grade clinical symptoms. *C. acnes* has been thought to escape the immune system at bone sites.

**Material and methods:**

Our study was carried out on a laboratory strain and two BPI-related clinical strains, one of which surprisingly induced clinical symptoms of inflammation in the patient. We investigated the ability of these *C. acnes* strains to trigger *in vitro* human primary neutrophils (PMN) response through inflammatory mediators measurements (antibody arrays, ELISA, RT-qPCR, zymography) and activation status assessment (flow cytometry), and to induce *in vivo* PMN recruitment from the bloodstream in mice air-pouch model. PMN-mediated inflammation was also studied in an original *in vitro* model mimetic of an infected bone site that combine titanium alloy, human primary osteoblasts, human primary neutrophils and *C. acnes* strains.

**Results:**

We demonstrated for the first time that both *C. acnes* planktonic and biofilm cultures, triggered an effective immune response by neutrophils *in vitro* and their recruitment *in vivo*. This host response was enhanced when using a strain from a patient with inflammatory signs. In an original infected prosthesis mimetic model, osteoblasts and neutrophils were able to detect *C. acnes*, but their response to the clinical *C. acnes* inflammatory strain decreased.

**Conclusion:**

This work provides the first evidence showing that the immune cell response to pathogenic *C. acnes* may be tuned by nonimmune cells at the infected site, such as osteoblasts, which may promote bacterial persistence.

## 1 Introduction

*Cutibacterium acnes* is a commensal microorganism of human skin that is known for its involvement in the inflammatory process of acne (1). However, this species is also recognized as the causative agent of various types of implant-associated infections (2). *C. acnes* is identified in at least 10% of bone prosthesis infections (BPIs) (3), among which 56% are shoulder prosthesis infections (4). The slow growth of this species is an issue because of non-adapted diagnostic methods and culture time (5). The need for specific bacteriological culture conditions along with low-grade clinical symptoms accounts for the complicated and delayed diagnosis and treatment of such infections (2).

Various studies on acne vulgaris have underscored the ability of *C. acnes* to interact with immune cells through different pathways to promote inflammation (6). *C. acnes* triggers the secretion of inflammatory cytokines by immune cells in acne through toll-like receptor (TLR) or inflammasome pathway activation (7, 8). The inflammatory cascade induced by bacteria is also the primary cause of many other chronic diseases, such as sarcoidosis, prostate inflammation leading to cancer, intervertebral disc degeneration, and Synovitis, Acne, Pustulosis, Hyperostosis, Osteitis (SAPHO) syndrome (9). In such contexts (i.e., disc degeneration), studies have shown *ex vivo* that *C. acnes* infection may promote inflammation *via* the secretion of proinflammatory mediators such as TNF-α, IL-8, IL-6 and MCP-1, which are associated with the accumulation of neutrophils and Modic scale changes (10, 11). In contrast, *C. acnes* does not induce a clear inflammatory response at bone sites of infection. Patients with *C. acnes*-related BPIs present with few clinical symptoms, which typically manifest 3 to 24 months after surgery, unlike early symptomatic infections, such as *Staphylococcus aureus* infections, which occur within the first 3 months and lead to obvious signs of inflammation, including redness or fever (12). In *C. acnes*-related BPIs, the levels of serum inflammatory markers are often not increased (13), and anatomical pathology examination of the tissues surrounding the prosthesis does not reveal acute or chronic inflammation. Hence, researchers thought that the microorganism evaded the immune system. Putative mechanisms may include bacterial internalization by host cells (14) and biofilm formation. The latter promotes virulence and may explain the persistence of bacteria in the context of a *C. acnes*-related BPI, as it confers considerable resistance to host defenses and tolerance to antimicrobial agents (15–17).

The implantation of a biomaterial induces a foreign body reaction with establishment of the innate immune response, which elicits the recruitment of immune cells, including polymorphonuclear neutrophils (PMNs), followed by monocytes and lymphocytes. PMNs represent 50% to 70% of total circulating leukocytes and are considered the first line of host defense (18). They display various antimicrobial functions, including degranulation, phagocytosis, the production of reactive oxygen species, and the formation of neutrophil extracellular traps (NETs) (19, 20). To eradicate microorganisms, neutrophils enlist their cytoplasmic granules, starting with secretory granules and tertiary granules, which are associated with an increase in CD16 receptor expression. Then, the exocytosis of secondary followed by primary granules leads to an increase in the surface expression of CD66b and CD63 and final shedding of the CD16 receptor by the released proteases (21, 22). In response to various stimuli, neutrophils can also produce cytokines and other inflammatory factors with immunomodulatory properties bestowing to neutrophils a pivotal role in the entire immune system. Ultimately, to grapple bacteria, PMNs can release chromatin filaments, called NETs, into the extracellular space (23).

The purpose of this study was to decipher the inflammatory processes triggered by *C. acnes* in the bone microenvironment to better understand the detection of *C. acnes* by innate immune cells in the context of *C. acnes*-related BPIs. A laboratory strain (CIP) and two BPI-related clinical strains (BPI and BPI infl) were obtained from patients with two distinct clinical inflammatory profiles. The interaction of *C. acnes* clinical strains with human primary neutrophils was investigated *in vitro*, focusing on the ability of bacteria to induce the release of proinflammatory markers, immune cell activation and cell death, both in biofilms and in the planktonic state. Then, the *in vivo* response of PMNs to planktonic *C. acnes* strains was observed in a mouse air pouch model. Finally, in an original *in vitro* model mimicking an infected bone site, PMN-mediated inflammation was studied.

## 2 Materials and methods

### 2.1 Bacterial strains

Our work was conducted using three different *C. acnes* strains, one laboratory strain, CIP 53.117 (“Collection de l’Institut Pasteur” ATCC 6919), which was isolated from a patient with facial acne, and two clinical strains, which were isolated at the bacteriology laboratory of Reims University Hospital from a bone prosthesis infection (NCT03950063). The first clinical strain, named BPI infl, induced an increase in circulating inflammatory biomarkers in the patient (C Reactive Protein, leukocytes), which is rare, and the second, called BPI, did not induce inflammatory markers (**Supplementary Table 1**).

### 2.2 Whole genome sequencing and bioinformatic analysis

Whole-genome sequencing of our three strains was performed using Illumina short-read technology (DNA prep kit for library preparations and NextSeq sequencer) with a read length of 2×150 bp. Adaptors were removed using cutadapt v3.4, and reads were trimmed using trimmomatic v0.39 with the options ‘LEADING:3 TRAILING:3 SLIDINGWINDOW:4:15 MINLEN:36’. SPAdes v3.13.0 was used for assembly with the options ‘-careful-cov-cutoff auto’. Assembly quality was assessed with QUAST v5.0.2. Annotation was performed with Prokka v1.14.6. For some genes, we used InterProScan v5.55-88.0 to predict the presence of protein domains. Pangenome analysis was conducted with Roary v3.13 to identify genes present in only the BPI infl strain. We searched for antimicrobial resistance or virulence genes with ABRicate v0.8.13 and the following databases: NCBI AMRFinderPlus, ARG-ANNOT, CARD, MEGARES, Resfinder and VFDB. Prophages were identified using the PHASTER web server. Plasmid Finder v2.0.1 was used to search for plasmid sequences. Bioinformatics analyses of the genomes of the three strains revealed that the BPI infl strain possessed four consecutive genes that are only present in its genome and not in the other two strains: the cell division protein FtsK, one site-specific recombinase belonging to a phage integrase family, and two hypothetical proteins (**Supplementary Figure 1**). MLST was determined using PubMLST. The MLST determination is based on the work by McDowell et al., 2012 at using the following genes for the determination: aroE, atpD, guaA, lepA, sodA, gmk, tly, camp2 (24).

### 2.3 Bacterial culture

*C. acnes* strains were isolated on Columbia agar with 5% sheep blood (Bio-Rad) under anaerobic conditions using a GENbag anaer system (Biomérieux) at 37°C. Planktonic *C. acnes* strains were cultivated in brain heart infusion (BHI, Bio-Rad) broth for four days (96 h) at 37°C. These precultures were washed in sterile Dulbecco’s phosphate buffered saline (DPBS), and optical density measurement at 600 nm was performed to adjust bacterial suspensions concentration to reach the targeted multiplicity of infection in Roswell Park Memorial Institute 1640 medium GlutaMAX™ supplement (RPMI) for interaction with PMNs or in Dulbecco’s modified Eagle’s medium GlutaMAX™ supplement (DMEM) for the tripartite model (all from Thermo Fisher Scientific). For biofilm formation, the optical density at 600 nm of *C. acnes* precultures was adjusted to 0.01 before seeding the bacteria in the wells containing Thermanox^®^ plastic coverslips (Thermo Fisher Scientific). After five days (120 h) of growth at 37°C, biofilms formed on coverslips were gently washed in BHI to remove planktonic bacteria and transferred to new wells containing RPMI medium.

### 2.4 Collection of blood samples, cell isolation and culture

Venous blood was collected with EDTA from healthy donors of the “Établissement Français du Sang Grand Est” (Authorization ALC/PIL/DIR/AJR/FO/606 Reims, France) with the written informed consent of the donors following ethical and legal regulations (Article R1243-57), in accordance with the authorization and registration number DC-2014-2262 provided by the French Ministry. A total of 112 donors aged between 20 and 67 years (average age 48) were included, 24% of which were women and 76% of which were men. Polymorphonuclear neutrophils were purified from whole human blood using Polymorphprep™ (ProteoGenix). The residual erythrocytes were removed by hypotonic shock. PMNs were resuspended in RPMI medium supplemented with 2.5% heat-inactivated autologous human serum and counted with a Neubauer chamber (Hirschmann). One million PMNs were cultured in the presence of different *C. acnes* strains. Two negative controls, one with cells without bacteria and the other containing culture medium alone, were systematically tested to determine the initial inflammatory state of the donor cells. A positive control for inflammation, containing cells stimulated with 100 ng/mL LPS from *E. coli* O111:B4 (Sigma Aldrich), was also used to ensure that the cells could respond to inflammatory stimuli (25). PMN/*C. acnes* interactions were carried out in 24-well plates with one million cells in 1 mL, and 120 h bacterial biofilms for one experiment and 96 h planktonic bacteria at a multiplicity of infection (MOI) of 20:1 and 2:1 for the other experiment; both experiments were conducted for 4 h at 37°C in a humidified atmosphere with 5% CO_2_. After incubation, the contents of the wells were collected and centrifuged (600 g, 20°C, 10 min). The cell pellets were dry-frozen at -80°C, and after centrifugation at 6000 g, the clarified supernatants were frozen at -20°C for further analyses.

### 2.5 Cytokine antibody array

Cytokine antibody arrays (“Human Cytokine Array C5”, RayBioTech^®^ C-Series) were performed after 4 h of interaction according to the manufacturer’s instructions. One-half of the culture supernatants diluted with blocking buffer were incubated on antibody-coated membranes before detection with a streptavidin-horseradish peroxidase biotinylated-antibody complex and chemiluminescence detection. The integrated optical density (IOD) of each dot was measured with ImageJ software Fiji V1.54f after image acquisition on a Bio-Rad ChemiDoc™ MP. The ratios of the values obtained in the *C. acnes*-treated group compared to those in the PMN control group were calculated to determine the effect of *C. acnes* on cytokine production. Variations in cytokine production were considered when the variation was revealed in at least four of the five independent PMN donors. Threshold values were determined based on the maximum variation detected in the negative controls. Only upward variations greater than 1.49 and downward variations less than 0.43 were reported.

### 2.6 Enzyme-linked immunosorbent assay

Human IL-8, TNF-α, IL-6, MIP-1β and MCP-1 concentrations in interaction supernatants and murine KC and TNF-α in air pouch exudates were measured using ELISA (Duoset, R&D Systems) kits following the manufacturer’s instructions. Protein concentrations were estimated using a range of standard concentrations of recombinant proteins. The controls included non-stimulated cells and medium alone. Absorbance measurements were performed at 450 nm and corrected to 570 nm using a FLUOstar Optima^®^ microplate reader (BMG Labtech).

### 2.7 Gelatin zymography

Gelatin zymography was used to measure matrix metalloproteinase-9 (MMP-9)-related gelatinolytic activity in conditioned supernatants. The total protein concentrations of the supernatants were measured *via* absorbance at 280 nm with a Nanodrop™ 2000c spectrophotometer (Thermo Fisher Scientific), and equal amounts of protein (4 µg) were used for zymography experiments. The negative control supernatants (cells alone and culture medium alone) and positive control supernatants (cells stimulated with LPS) were systematically deposited on each gel. A control lane containing 20 µL of recombinant human MMP-9 (Sigma Aldrich) (at a final concentration of 50 µL/mL) was also used. Gelatinolytic activities in the supernatants were evaluated on 10% SDS−polyacrylamide gels containing 1 mg/mL gelatin (Sigma Aldrich) as described in our previous work (25). The migration was carried out for 30 min at 80 V in the compression gel and then at 160 V in the separation gel using a Bio-Rad Power Pac 200. After electrophoresis, SDS (Bio-Rad) was removed from the gel by two incubations in 2.5% Triton X-100 (Thermo Fisher Scientific) for 30 min at room temperature. Then, the gels were incubated for 18 h at 37°C in 50 mM Tris-HCl (pH 7.6), 0.2 M NaCl and 5 mM CaCl_2_ (all from Sigma Aldrich). The gels were stained for 30 min with Coomassie Blue G-250 (Bio-Rad) and then destained for 2 × 30 min in 10% methanol and 20% acetic acid (VWR Chemicals). Gel digitalization was performed on a Vilber Lourmat CN-UV/WL system controlled by BioCapt^®^ software v12.6. Proteolytic activities were determined based on clear bands against the blue background of stained gelatin and quantified as the integrated optical density (IOD) by ImageJ software Fiji V1.54f. Three forms of MMP-9 were detected and quantified: MMP-9 (92 kDa), MMP-9 complexed with lipocalin (135 kDa) and MMP-9 homodimers (235 kDa) (26).

### 2.8 RNA purification, reverse transcription, and RT−qPCR

Total RNA was extracted from cell pellets with a MasterPure™ RNA purification kit (Biosearch Technologies) according to the manufacturer’s protocol. Total RNA was reverse-transcribed into cDNA using an Eppendorf^®^ Nexus GSX1 Mastercycler (Thermo Fisher Scientific) and a high-capacity cDNA reverse transcription kit (Thermo Fisher Scientific) according to the manufacturer’s instructions. Primers were designed for the *RPS18* (internal reference), *CXCL8*, *TNFA*, *MMP9*, *IL6*, *CCL2*, and *CCL4* genes (Eurogentec) (**Supplementary Table 2**). Transcription products were amplified by qRT−PCR on a StepOnePlus™ Real-Time PCR System (Applied Biosystems). Takyon™ Rox SYBR^®^ MasterMix (Eurogentec) was used for amplification. After a first denaturation step at 95°C for 10 min, qRT−PCR was performed according to a thermal program with 40 cycles of denaturation at 95°C for 15 sec, followed by annealing and extension at 60°C for 1 min. Data collection was performed at the end of each annealing/extension step. The third step consisted of a dissociation process to ensure the specificity of the amplicons by measuring their melting temperature (Tm). Data analysis was performed with StepOne™ Software v2.3 (Applied Biosystems).

### 2.9 Scanning electron microscopy

Scanning electron microscopy (SEM) was performed after 1 and 4 h of direct PMN/*C. acnes* interaction to examine cell and bacterial morphology, as well as NET production. SEM was also used in the tripartite model to visualize *C. acnes*-infected osteoblasts after 24 h of incubation, as well as after 4 h of OB/*C. acnes*/PMN interaction. The supernatants were removed, and the samples were fixed with 2.5% glutaraldehyde (Sigma Aldrich) in 1× DPBS for 1 h at 4°C. After two washes for 10 min in distilled water, the cells were dehydrated in a graded alcohol solution bath (50%, 70%, 90% and 100% two times) for 10 min (Charbonneaux-Brabant). The samples were finally desiccated with hexamethyldisilane (HMDS, Sigma Aldrich) for 5 min. The samples were sputtered with a thin gold-palladium film using a JEOL ion sputter JFC-1100 instrument (8 mA and 1200 V). The cells were observed using a Schottky field emission scanning electron microscope (JEOL JSM-7900F) with a primary beam energy of 2 kV.

### 2.10 Annexin V/propidium iodide staining

Neutrophil apoptosis induced by planktonic bacteria was assessed with Annexin V (AV)-FITC/propidium iodide (PI) labeling and flow cytometry analysis (FITC Annexin V Apoptosis Detection Kit, Thermo Fisher Scientific). After 4 h of incubation, the contents of the culture wells were collected, and the cell pellets were stained according to the manufacturer’s instructions and subjected to flow cytometry with a BD LSR Fortessa™ system (BD Biosciences). AV-FITC was excited with a 488 nm excitation laser, and PI was excited with a 561 nm excitation laser. The emission filters used to detect fluorescence signals were 530/30 nm for AV-FITC and 610/20 nm for PI. We then used the analysis strategy described in our previous work (27). In the first stage, cells were selected by an FSC-A/SSC-A gate to exclude subcellular debris. In this population, single cells were selected by an SSC-H/SSC-W gate. Apoptotic cells were then identified by an AV/PI gate. These regions were discriminated using unstained and mono-labeled stained controls. Ten thousand cells were collected per sample. Data analysis was performed with FlowLogic software 700.0A (Inivai™ Technologies). AV^+^PI^-^ cells were considered early apoptotic cells.

### 2.11 LDH measurement

Lactate dehydrogenase (LDH) activity was evaluated in neutrophil-conditioned media as a marker of cell death with a cytotoxicity detection kit (Sigma Aldrich) according to the manufacturer’s instructions. The absorbance was measured at 490 nm with correction at 700 nm using a BMG Labtech FLUOstar Optima^®^ microplate reader.

### 2.12 Phagocytosis assay

Following a 1 h incubation of PMN/planktonic bacteria, the internalized bacteria and those attached to the PMN membrane were separated from the free bacteria present outside the cells using a CD66abce MicroBead kit (Miltenyi Biotec). After magnetic labeling of the neutrophils, the samples were passed through elution columns in a magnetic field. The first elution with DPBS eliminated free bacteria remaining in the culture medium while neutrophils were retained in the column. The column was removed from the magnetic support, the magnetically labeled cells were flushed with DPBS, and the number of viable bacteria was assessed after Triton cell lysis (0.1% Triton X-100). The lysates were serially diluted and plated on Columbia agar plates with 5% sheep blood to determine the number of bacteria.

### 2.13 PMN activation

To evaluate neutrophil degranulation in response to *C. acnes*, one million cells were collected after incubation for 4 h and stained with FVS780 (BD Biosciences) for 15 min. After nonspecific sites were blocked with a human Fc block™ (BD Biosciences), the cells were labeled with BV421 mouse anti-human CD66b (clone G10F5), PE mouse anti-human CD63 (clone H5C6) and PerCP-Cya5-5 mouse anti-human CD16 (clone 3G8) for 30 min (BD Biosciences). After washing with DPBS-EDTA, the cells were fixed in BD Phosflow™ Lyse/Fix Buffer 1X (BD Biosciences) and subjected to flow cytometry with a BD LSRFortessa™ system. FVS780 was excited with a 640 nm laser and detected with an emission filter 780/60. CD66b BV421 was excited with a 405 nm laser and detected with an emission filter at 450/50 nm. CD63 PE was excited with a 561 nm laser and detected with a 585/15 nm emission filter. CD16 PerCP-Cya5-5 was excited with a 488 nm laser and detected with a 710/50 nm emission filter. Data analysis was performed with FlowLogic software 700.0A.

### 2.14 *In vivo* air pouch model

Seven-week-old male and female BALB/c mice were obtained from Janvier Laboratories. Experiments were performed on mice housed in a controlled environment (temperature: 21 ± 2°C, relative humidity: 65 ± 15%, natural alternating light/dark cycle, (agreement n°B514543) in accordance with UE Directive 2010/63/EU, and the protocols approved by the Regional Ethics Committee on Animal Experimentation (CEEA n°056) and the Ministry of Agriculture, under the direction of investigators certified for animal experiments following the protocol APAFIS#38425-2022091013031605. Mice were acclimated for 1 week before air pouch induction. Before each injection, the mice were anesthetized using 5% isoflurane (Isoflu-Vet, Dechra). On day 0, 3 mL of 0.22 µm of filtered air was injected subcutaneously into the middle of the back of mice with a 10 mL syringe and 27 G needle (Terumo). On day 3, the injection was repeated. On day 7, inflammation was induced by injecting 2 mL of LPS (10 ng/mL) or 2 mL of bacterial suspension (10^6^, 10^7^, or 10^8^ bacteria). Control mice were injected with 2 mL of DPBS only. After 6 h, 24 h and 48 h, intracardiac blood was collected under gaseous anesthesia before the animals were sacrificed. To remove the exudate from the air pouch, 2 mL of 5 mM DPBS-EDTA was injected, and after 30 sec of soft massage of the pouch, the exudate was collected. Cells from the exudate were washed and counted using a Kova^®^ slide.

### 2.15 Immune cell recruitment in air pouch

To evaluate leukocyte subsets and discriminate neutrophils, 300,000 cells were collected per air pouch exudate sample and then stained with FITC rat anti-mouse Ly-6G (clone 1A8) and BV421 rat anti-mouse F4/80 (clone T45-2342) (BD Biosciences). After a wash, the cells were fixed in BD Lyse/Fix and subjected to flow cytometry with a BD LSR Fortessa™ system. F4/80-BV421 was excited with a 405 nm laser and detected with an emission filter at 450/50 nm. Ly-6G-FITC was excited with a 488 nm excitation laser and detected with an emission filter at 530/30 nm. In the first stage, the cells were filtered with an SSC-A/FSC-A gate. In this population, single events were filtered by an FSC-A/FSC-H gate to exclude subcellular debris. Then, the PMNs were identified as Ly-6G-PE^+^ F4/80-BV421^-^ cells. Data analysis was performed with FlowLogic software 700.0A.

### 2.16 Histological analysis

For histological analysis, collected air pouches were fixed in 4% formaldehyde (VWR Chemicals) for one week, washed and dehydrated by gradually replacing water in the sample with alcohol; thereafter, the alcohol was replaced with xylene. The samples were then embedded in paraffin, and multiple serial 5 µm thick sections were obtained. Sections were rehydrated and subjected to Masson’s trichrome staining (hematoxylin, fuchsin and aniline blue). Multiple histochemical analyses were carried out on serial sections to identify neutrophils and *C. acnes*. The samples were blocked with Bloxall^®^ Endogenous Blocking Solution (Vector Laboratories) and stained using a primary recombinant rabbit anti-mouse Ly-6G antibody (clone EPR22909-135, Abcam) (1:4000 in 1% BSA) and a secondary goat anti-rabbit antibody coupled to streptavidin-AlexaFluor^®^568 (1:50 in 1% BSA) (Thermo Fisher Scientific). The samples were incubated overnight with a mouse anti-*C. acnes* monoclonal antibody at 0.5 µg/mL (clone TMDU2, MBL bio). After rinsing, the samples were incubated for 30 min with a biotinylated horse anti-mouse secondary antibody (diluted at 1/200 in DPBS/BSA 1%) (Vector Laboratories). Streptavidin-AlexaFluor^®^488 (1:2000 in 1% BSA) (Thermo Fisher Scientific) was then added to bind biotin, and finally, nuclear staining was performed with DAPI (1:3000 in distilled water) (Thermo Fisher Scientific). Sections were then mounted with Dako Fluorescent Mounting Medium (Agilent) and a coverslip, and observations were performed using a Zeiss Axiovert 200 M inverted microscope and coupled AxioVision™ v2.8 software.

### 2.17 *In vitro* mimetic model of bone prosthesis infection

Shot-peened titanium supports (Ti-6Al-4V, 1.13 cm²) were placed in an acetone bath, ultrasonicated for 15 min, and then sterilized by dry heat in a Poupinel oven (Memmert) for 2 h at 250°C. Human primary osteoblasts (OBs) were obtained from the femoral heads of patients in the Orthopedic and Traumatology Department of the Reims University Hospital (CHU Reims), as described previously (28). Samples were collected after written informed consent had been given by donors following ethical and legal regulations (Article R1243-57), in accordance with the authorization and registration number DC-2014-2262 given by the French Ministry. The explants were cut into small pieces, washed in DPBS four times for 5 min, and digested in a solution of 0.5% trypsin, 5.3 mM EDTA (Sigma Aldrich), and then in type II collagenase (1.4 mg/mL, Thermo Fisher Scientific). The fragments obtained were thereafter placed in 25 cm² culture flasks containing DMEM supplemented with 10% fetal bovine serum (FBS, PAN Biotech) and 1% penicillin−streptomycin (Thermo Fisher Scientific) and then incubated at 37°C in a 5% CO_2_ humidified atmosphere. The cells were amplified for four passages.

Primary osteoblasts were seeded in drops on titanium (20 000 cells) in DMEM supplemented with 10% FBS and 1% penicillin/streptomycin and incubated at 37°C and 5% CO_2_ for four days to reach an average of 100,000 cells. The day before infection, the medium was replaced with DMEM supplemented with 10% FBS to remove any trace amounts of antibiotics. Osteoblasts were infected with two *C. acnes* strains, CIP and BPI infl, for 3 h (MOIs of 20:1 and 200:1). The initial amount of bacteria was evaluated by plating dilutions on sheep blood plates under anaerobic conditions. After 3 h of infection, the cells were washed with DPBS and incubated for 1 h with medium containing 1% penicillin/streptomycin. Osteoblasts were washed again with DPBS and incubated for an additional 24 h in DMEM supplemented with 10% FBS. Osteoblast supernatants were collected for further analysis. One million PMNs were obtained from whole blood as described above and resuspended in 500 µL of RPMI medium supplemented with 5% heat-inactivated autologous human serum; then, PMNs were cultured with osteoblasts infected or not infected with *C. acnes* in 500 µL of osteoblast medium for 4 h. PMNs resuspended in 500 µL of RPMI medium supplemented with 5% heat-inactivated autologous human serum were challenged with 500 µL of infected OB-conditioned supernatants. After incubation, the final supernatants were collected, and titanium supports were prepared for SEM as described above or immunolabeled as described in the following section.

### 2.18 Tripartite interaction immunofluorescent staining

Titanium supports were rinsed with DPBS, fixed with 4% paraformaldehyde (Thermo Fisher Scientific) for 10 min at 37°C, and permeabilized with 0.5% Triton X-100 for 15 min at room temperature. After 3% BSA saturation for 30 min, the samples were incubated for 1 h with anti-*C. acnes* monoclonal antibody (0.25 µg/mL) (clone TMDU2, MBL bio). Then, the samples were incubated for 30 min with biotinylated anti-mouse secondary antibody (diluted at 1/200 in DPBS/BSA 1%) (Vector Laboratories), rinsed in DPBS, and incubated with streptavidin-Alexafluor^®^488 (diluted at 1/3000) (Thermo Fisher Scientific) for 30 min. The actin filaments were identified after a 30 min incubation in phalloidin-AlexaFluor^®^568 (diluted 1/100 in DPBS/BSA 0.5%) (Thermo Fisher Scientific), and the nuclei were then visualized with DAPI (1/3000 in distilled water) (Thermo Fisher Scientific) for 5 min. Finally, a glass coverslip was mounted with the fluorescent mounting medium Dako on the titanium studs, and the samples were observed *via* confocal laser scanning microscopy (CLSM) (LMS880 Airy Scan CLSM, Zeiss).

### 2.19 Statistical analysis

Each *in vitro* culture modality was performed on cells from at least ten independent human donors unless otherwise stated, and *in vivo* tests included 6 mice per group. The significance of the results was assessed with the nonparametric Kruskal-Wallis test followed by *post hoc*, exact (stratified for blood donors) nonparametric Wilcoxon Mann-Whitney tests (StatXact 7.0, Cytel Inc.). We used nonparametric statistics because there was not a normal distribution of the assessed variables. Stratification allowed the impact of donor variability to be considered. Differences were considered significant at *p*<0.05. In the graphic representation, the red bars represent the median values, the black bars represent the 1^st^ and 9^th^ deciles, the limits of the rectangles represent the 1^st^ and 3^rd^ quartiles, and the triangles represent the average values.

## 3 Results

### 3.1 *C. acnes* biofilms activate and damage PMNs

**Figure 1:**
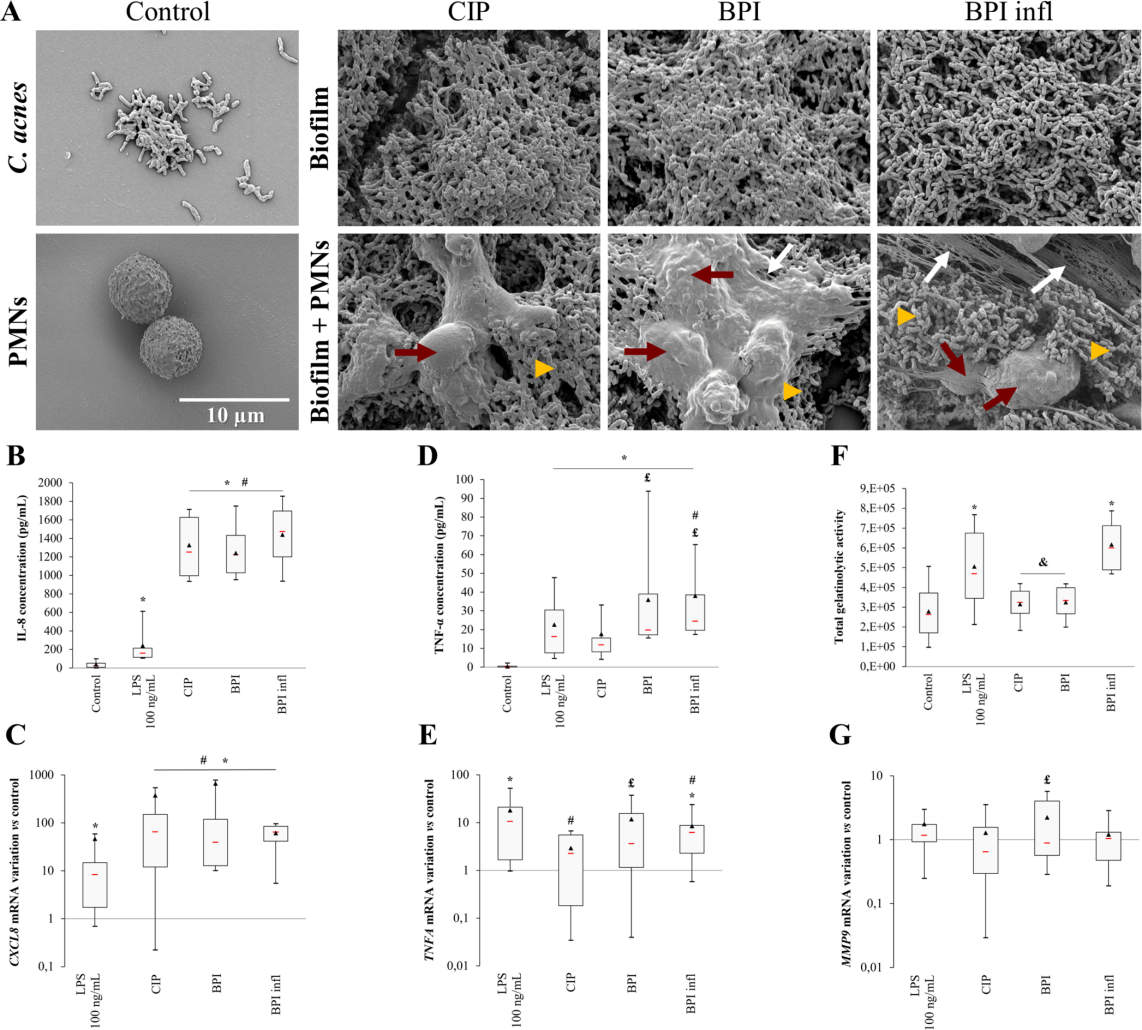
*C*. *acnes* biofilms damage PMNs and induce a strong PMN response. **(A)** Representative (n=4 independent PMN donors) SEM images of CIP, BPI and BPI infl 120 h biofilms without (upper line) or with (lower line) PMNs after 4 h of interaction (magnification ×5000, yellow triangles indicate bacteria, red arrows show PMNs, white arrows indicate NETs). **(B)** IL-8 concentration in culture supernatants and **(C)** *CXCL8* gene expression, **(D)** TNF-α concentration and **(E)** *TNFA* gene expression, **(F)** MMP-9-related gelatinolytic activity and **(G)** *MMP9* gene expression after allowing PMNs and *C*. *acnes* 120 *h* biofilms to interact for 4 h. **(B, D)** Data were obtained by ELISA. **(F)** Data were collected from gelatin zymography gels. **(C, E, G)** mRNA data were collected from qRT-PCR experiments and expression was determined according to the 2^-ΔΔCt^ method; these data were compared to control conditions. RPS18 was used as an internal reference *p<0.05 *vs.* control, #*p*<0.05 *vs.* LPS, £*p*<0.05 *vs.* CIP, &*p*<0.05 *vs.* BPI infl, n=10 independent PMN donors.

As the clinical diagnosis of a *C. acnes*-related BPI is mainly associated with the presence of a biofilm on the implant, we first focused on cell behavior during PMN/biofilm interactions. Scanning electron microscopy (SEM) analysis of biofilms revealed that the different *C. acnes* strains had similar morphologies. Biofilms were thick with little matrix. Bacteria seemed to aggregate, and few supposedly dead bacteria were found (**Figure 1A**). After letting cells interact with the biofilm for 4 h, images revealed that PMNs had a spread-out morphology compared with that of PMNs alone, whereas the morphology of the bacteria seemed unchanged following the addition of PMNs. We were able to image NET-like structures, especially in BPI infl samples.

### 3.2 *C. acnes* biofilms increase proinflammatory cytokine expression and release and matrix metalloproteinase activity in PMNs

To evaluate the response of PMNs to the infectious stimulus of *C. acnes* biofilms, the levels of major chemotactic (IL-8) and proinflammatory (TNF-α) mediators secreted by PMNs were determined by ELISA after incubating PMNs and *C. acnes* biofilms for 4 h. Compared with PMNs alone, *C. acnes* biofilms significantly increased IL-8 secretion by PMNs, with a 65-fold increase in the median value and no significant difference between strains (**Figure 1B**). We observed that the mRNA expression pattern of *CXCL8* was consistent with the protein expression data (**Figure 1C**). There was a significant increase in TNF-α production with all strains compared to the control, and increased secretion with both BPI-related clinical strains than in the laboratory CIP strain (*p*<0.05) (**Figure 1D**). We also observed a similar pattern in terms of *TNFA* gene expression (**Figure 1E**). The MMP-9-related gelatinolytic activity was examined by gelatin zymography and was increased only in the presence of BPI infl when compared to the control (*p*<0.05) (**Figure 1F**). *MMP9* gene expression was not different than that of the control; thus, the increase in the BPI infl strain at the protein level was not observed at the mRNA level (**Figure 1G**).

According to these data, biofilms did not hinder the recognition of bacteria by PMNs. Thus, we investigated interactions with planktonic bacteria at two different multiplicities of infection (MOIs) to determine whether *C. acnes* was recognized prior to biofilm formation in BPIs.

### 3.3 Planktonic *C. acnes* are internalized by PMNs

**Figure 2:**
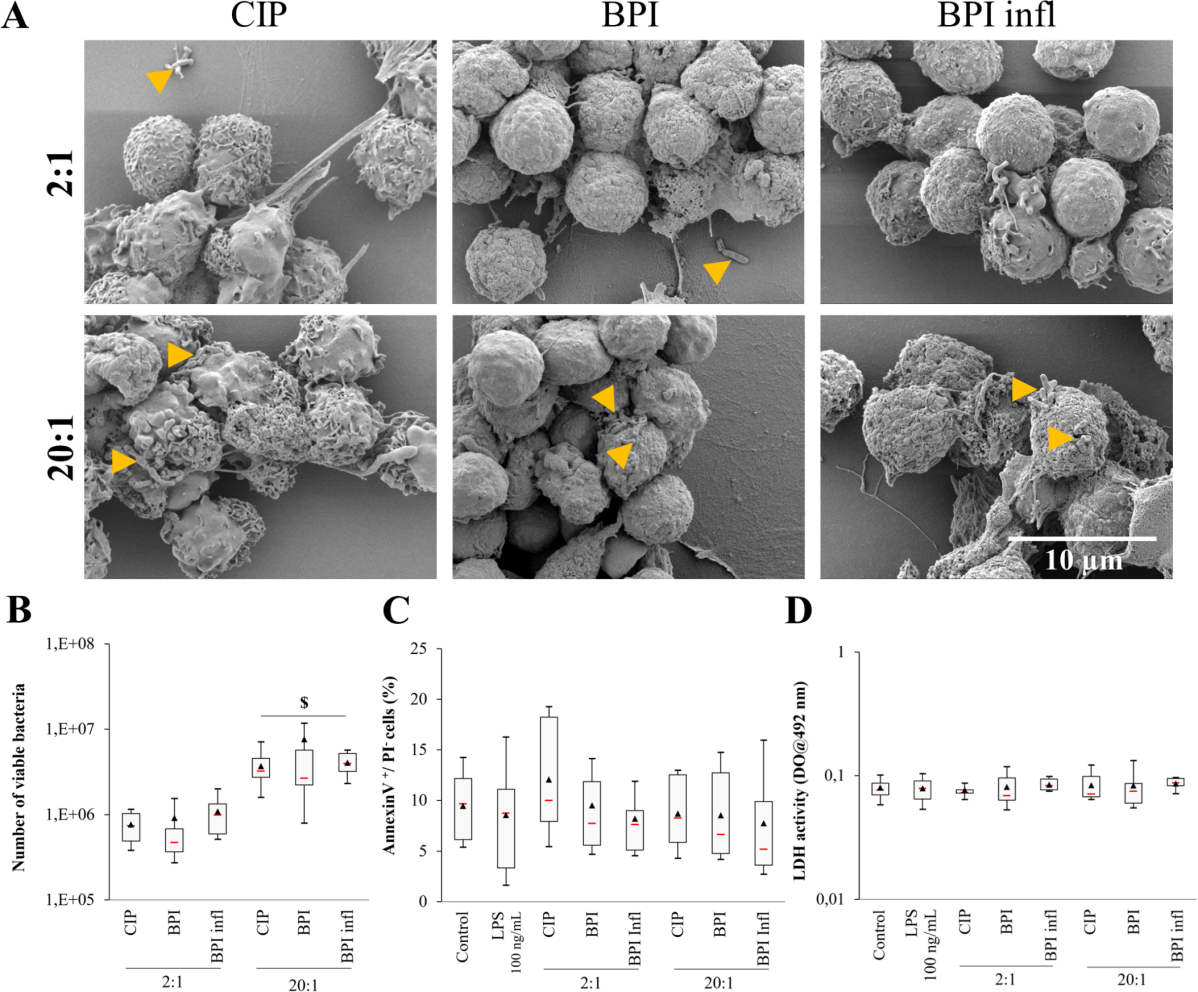
Planktonic bacteria are internalized by PMNs. **(A)** Representative (n=4 independent PMN donors) SEM images (magnification ×5000) of PMNs with CIP, BPI and BPI infl strains at MOIs of 2:1 and 20:1 after 1 h of interaction (yellow triangles indicate bacteria). **(B)** Viable internalized or attached bacteria after allowing PMNs and *C*. *acnes* strains at MOIs of 2:1 and 20:1 to incubate for 4 h. **(C)** Quantification of early apoptosis (AV^+^/PI^-^ cells) and **(D)** cell death (LDH activity in culture supernatants) after allowing PMNs and *C*. *acnes* strains at MOIs of 2:1 and 20:1 to incubate for 4 h. $*p*<0.05 between MOIs; n=10 independent PMN donors.

We found that the three *C. acnes* strains may be found inside PMNs or attached to their membrane (**Figures 2A, B**). Using SEM images, we identified planktonic bacteria attached to cells with more bacteria visible after 1 h compared to that after 4 h (**Figure 2A**, only images at 1 h are shown). SEM images also revealed an increase in the amount of bacteria stuck to cells at an MOI of 20:1. According to the phagocytosis assay results, significantly more live bacteria remained after we removed free bacteria at the highest MOI (*p*<0.05 between MOIs), but there was no variation between the different strains (**Figure 2B**). At both MOIs tested, planktonic *C. acnes* did not induce apoptosis or cell lysis during the interaction, as we did not observe any signal variation after Annexin V/PI labeling and flow cytometry analysis of PMNs, and the LDH activity in the supernatants was the same as that of the control (**Figures 2C, D**).

### 3.4 Planktonic *C. acnes* elicits the production of an MOI- and strain-dependent proinflammatory mediators by PMNs

**Figure 3:**
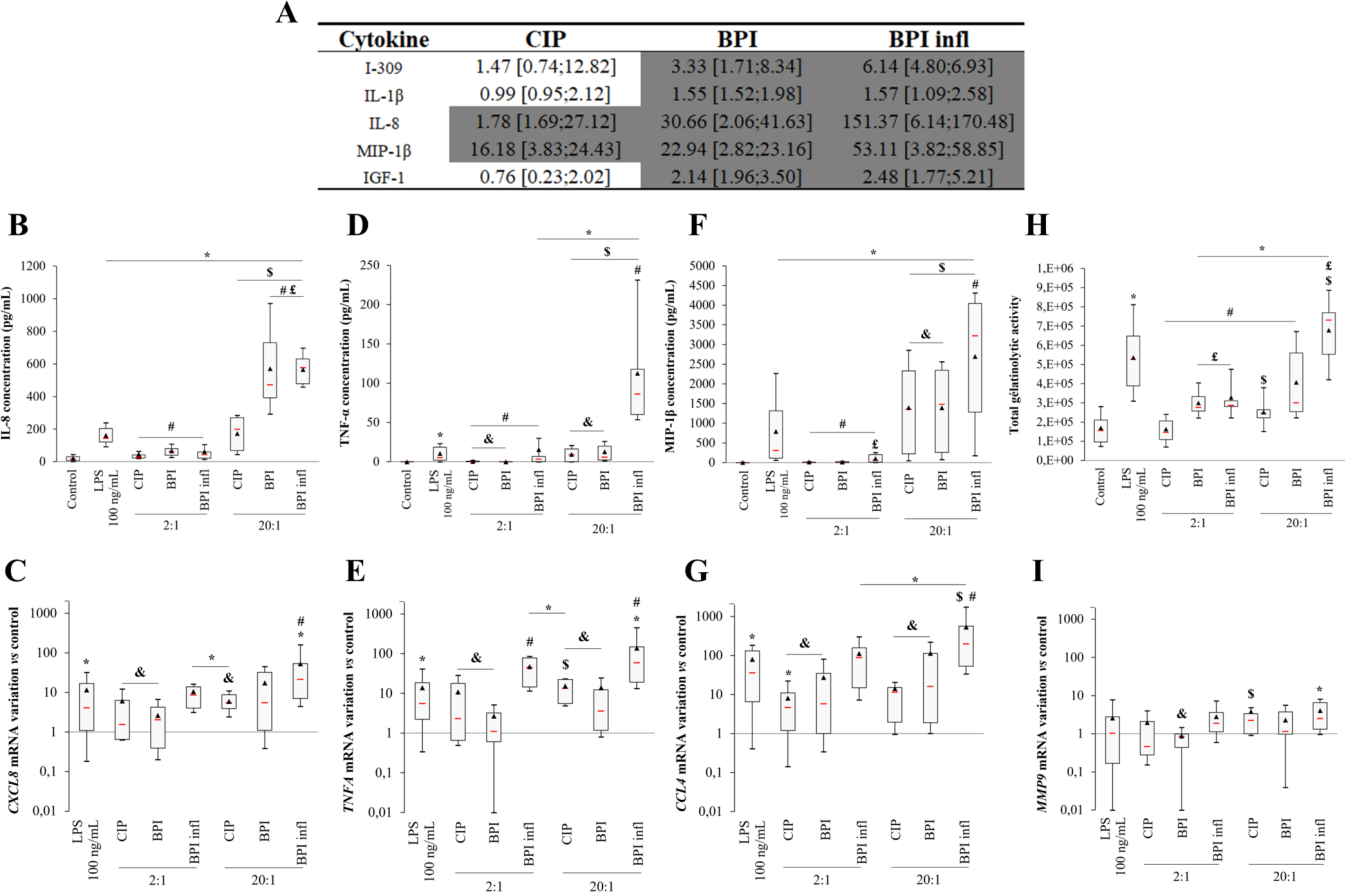
Planktonic *C*. *acnes* elicits MOI- and strain-dependent proinflammatory mediators production by PMNs. **(A)** Increased cytokine production induced by planktonic *C*. *acnes* strains, as measured by an antibody array. The ratio *versus* the control is presented as the median [1^st^ quartile; 3^rd^ quartile] for each cytokine evaluated. Values highlighted in dark gray indicate an increase of at least 1.49-fold compared with the control condition for at least 4 of 5 independent donors. **(B)** IL-8 concentration in culture supernatants and **(C)** *CXCL8* gene expression, **(D)** TNF-α concentration and **(E)** *TNFA* gene expression, **(F)** MIP-1β concentration and **(G)** *CCL4* gene expression, **(H)** MMP-9-related gelatinolytic activity and **(I)** *MMP9* gene expression after allowing PMNs and *C*. *acnes* strains at MOIs of 2:1 and 20:1 to incubate for 4 h. **(B, D, F)** Data were obtained by ELISA. **(H)** Data were collected from gelatin zymography gels. **(C, E, G, I)** mRNA data were collected from qRT-PCR experiments and expression was determined according to the 2^-ΔΔCt^ method; these data were compared to control conditions. RPS18 was used as an internal reference. *p<0.05 *vs.* control, #*p*<0.05 *vs.* LPS, $*p*<0.05 between MOIs, £*p*<0.05 *vs.* CIP, &*p*<0.05 *vs.* BPI infl, n=10 independent PMN donors.

The simultaneous screening of 80 human cytokines demonstrated that the production of proinflammatory mediators was induced by *C. acnes*. **Figure 3A** shows that the levels of five proinflammatory mediators increased with both BPI-related clinical strains (**Figure 3A**). This experiment allowed us to identify the more substantial variations and reinforced our interest in studying IL-8 secretion, as IL-8 was the mediator with the greatest increase (150-fold increase on average following incubation with BPI infl). MIP-1β, a CC chemokine, was also identified as a pertinent mediator, as it had highly upregulated expression when exposed to planktonic *C. acnes*, with a substantially increased response in the presence of BPI infl (50-fold increase on average). The other analyzed cytokines are shown in **Supplementary Table 3**. TNF-α was induced by the BPI infl strain (**Supplementary Table 3**) and remains a putative marker of interest in studying the PMN response to this specific strain. Notably, no MCP-1 or IL-6 production was found with this method.

The release of chosen proinflammatory mediators in culture supernatants obtained after 4 h of interaction was quantified precisely by ELISA. The interaction between PMNs and planktonic *C. acnes* induced a significant increase in the IL-8 concentration in culture supernatants in an MOI-dependent manner, regardless of the bacterial strain. BPI-related clinical strains triggered a more substantial PMN response at an MOI of 20:1- more than a 2-fold increase- than did the CIP strain (*p*<0.05) (**Figure 3B**). A similar trend was observed for *CXCL8* gene expression, with higher *CXCL8* expression in the BPI infl strain than in the CIP strain (**Figure 3C**). With the CIP and BPI strains, only an MOI of 20:1 promoted the production of the proinflammatory mediator TNF-α by PMNs. TNF-α production was significantly greater with BPI infl than with the other two strains, regardless of the MOI (**Figure 3D**). The *TNFA* gene expression pattern was consistent with the levels of secreted protein (**Figure 3E**). Significant MIP-1β production was observed in response to *C. acnes*, with an enhanced effect with the BPI infl strain at an MOI of 20:1 (**Figure 3F**). A similar trend was observed for *CCL4* gene expression (**Figure 3G**). Neither MCP-1 nor IL-6 secretion was detected in PMN-conditioned supernatants. No variation in *CCL2* and *IL6* gene expression was detected in *C. acnes* when compared with the control (data not shown). An increase in MMP-9-related gelatinolytic activity was observed in the presence of all bacterial strains at an MOI of 20:1 (**Figure 3H**). For strains associated with bone prosthesis infection, even a smaller amount of bacteria (MOI 2:1) was sufficient to induce a significant increase in MMP-9 gelatinolytic activity compared to that in the control (*p*<0.05). We did not observe any variation in *MMP9* gene expression compared to that in the control, except for a 2.49-fold increase in the median value with BPI infl at an MOI of 20:1 (**Figure 3I**).

### 3.5 Planktonic *C. acnes* triggers MOI- and strain-dependent PMN degranulation

**Figure 4:**
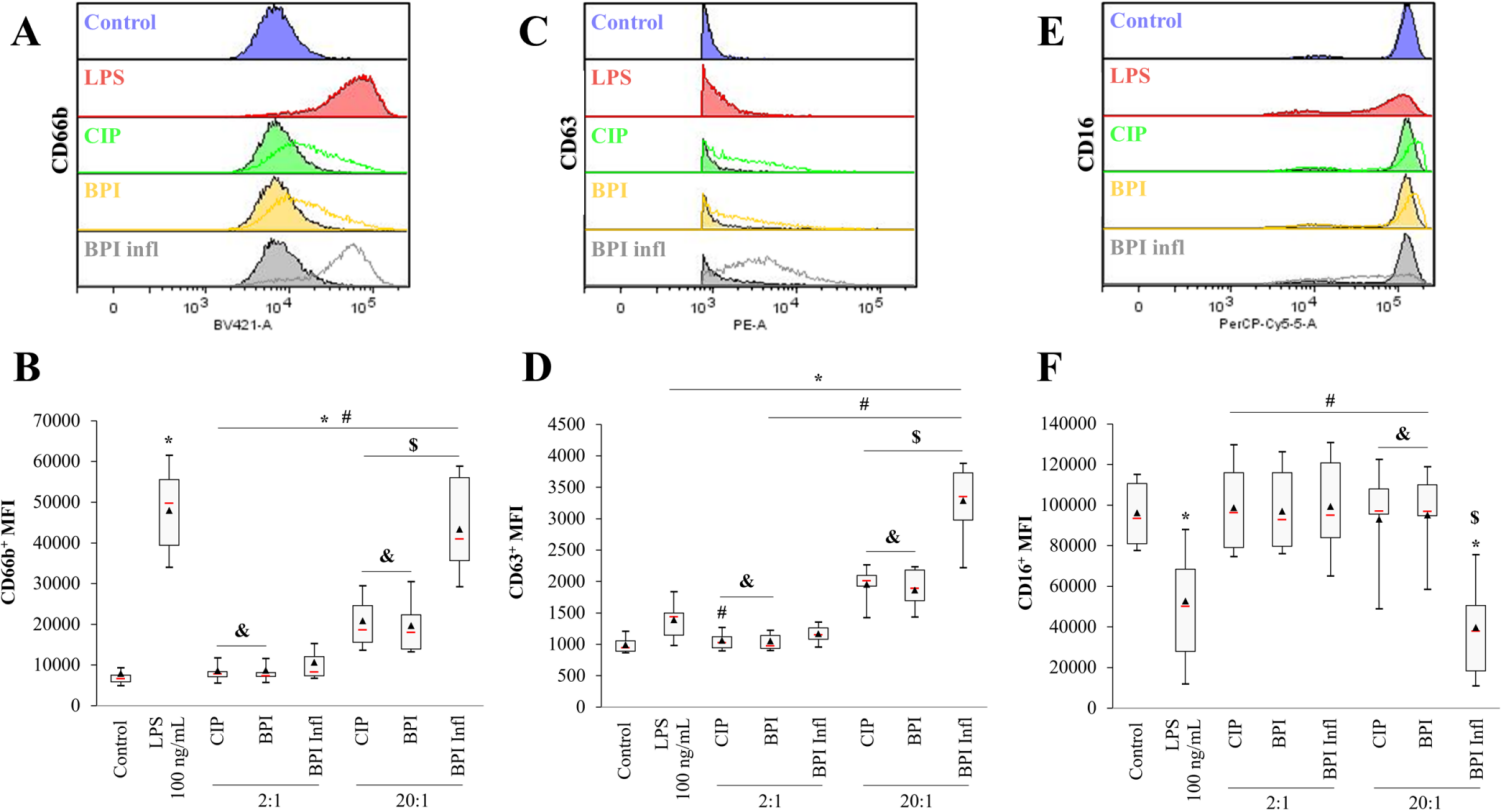
Planktonic *C*. *acnes* triggers MOI- and strain-dependent PMN degranulation. Representative histograms and quantification of **(A, B)** CD66b, **(C, D)** CD63, and **(E, F)** CD16 expression on the surface of PMNs after 4 h of incubation with *C*. *acnes* strains. The filled histograms represent an MOI of 2:1, and the empty histograms represent an MOI of 20:1. *p<0.05 *vs.* control, #*p*<0.05 *vs.* LPS, $*p*<0.05 between MOIs, £*p*<0.05 *vs.* CIP, &*p*<0.05 *vs.* BPI infl, n=10 independent PMN donors.

We further investigated the activation of neutrophils after interactions with bacteria. As CD66b, CD63 and CD16 are considered potent reporters of the degranulation process, these factors were labeled, and their expression was quantified by flow cytometry. All *C. acnes* strains induced a significant increase in CD66b and CD63 levels compared to those in the control. We observed a significant increase in the mean fluorescence intensity of CD66b (**Figures 4A, B**) and CD63 (**Figures 4C, D**) in PMNs with the BPI infl strain compared to that of other strains, and this increase was MOI-dependent (*p*<0.05). Conversely, with the BPI infl strain, there was a lower median fluorescence intensity of CD16 compared to the control and the other two strains at an MOI of 20:1 (*p*<0.05) (**Figures 4E, F**). Taken together, these results indicated increased PMN degranulation with BPI infl.

### 3.6 *C. acnes* induced PMN recruitment *in vivo*

**Figure 5:**
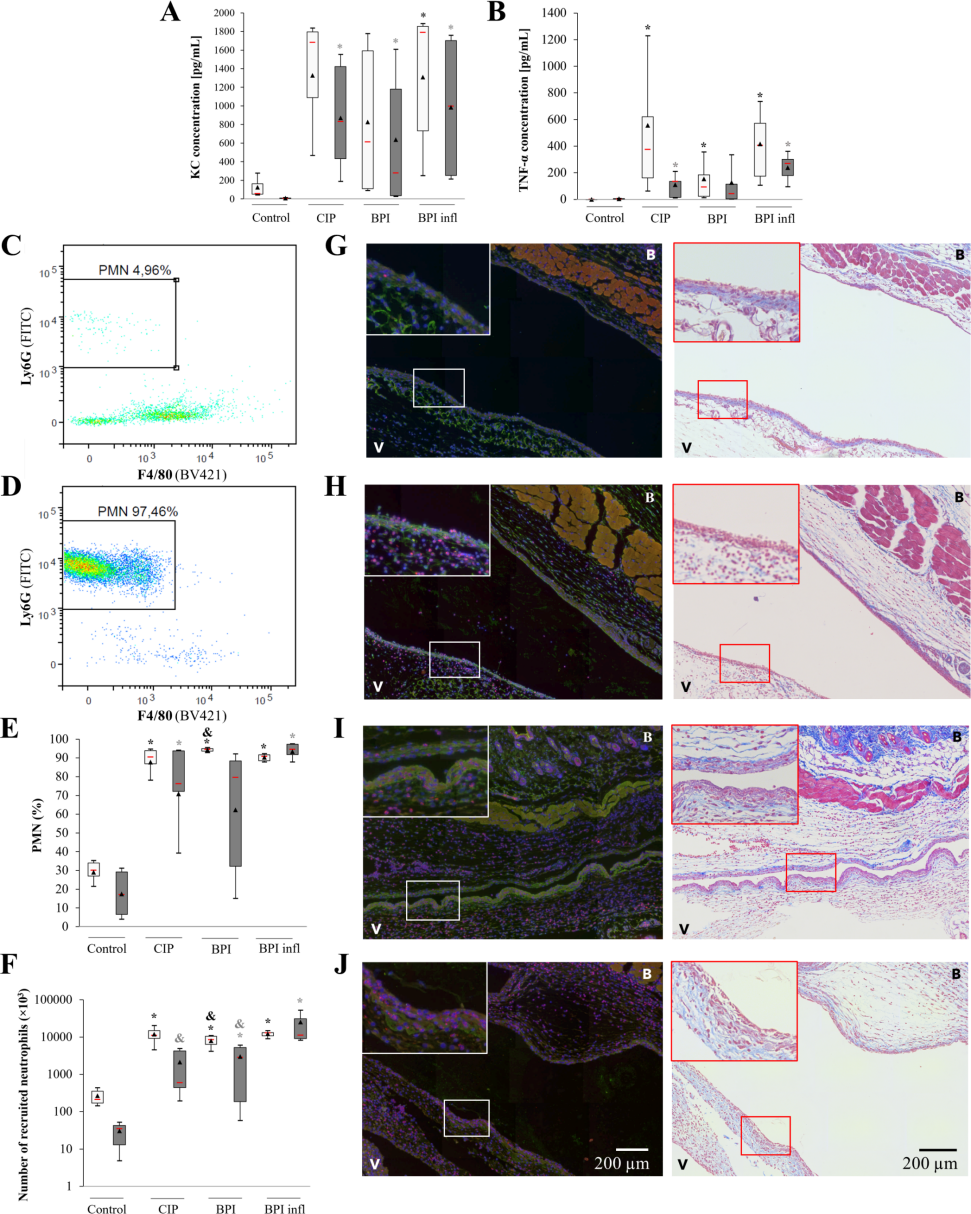
*C*. *acnes* strains induce PMN recruitment to the mouse air pouch. **(A)** KC and **(B)** mouse TNF-α concentrations in the air pouch exudates of female and male mice were measured by ELISA. Representative cytograms of the air pouch cell population in female mice **(C)** under control conditions and **(D)** after the BPI infl challenge for 6 h are shown. **(E)** The proportion and **(F)** number of PMNs recruited to the mouse air pouch 6 h after injection of PBS (control) or a *C*. *acnes* suspension (10^7^ bacteria) were determined by flow cytometry. Empty boxplots refer to male mice, and gray filled boxplots refer to female mice. *p<0.05 *vs.* control, #*p*<0.05 *vs.* LPS, &*p*<0.05 *vs.* BPI infl, n=6 mice per group. Representative immunofluorescence staining (nuclei in blue, Ly6G-positive PMNs in red, and *C*. *acnes* in green) and Masson’s trichrome-stained histological sections of air pouch membranes after 6 h of challenge with **(G)** PBS or **(H)** CIP, **(I)** BPI or **(J)** BPI infl suspensions (10^7^ bacteria). V: ventral side membrane, B: back side membrane. The insert at the top left of the microscopy images is an enlarged version of the framed portion. ×20 objective.

To assess the ability of *C. acnes* strains to induce PMN recruitment from the bloodstream *in vivo*, we used a murine air pouch model. Six hours after injection of the *C. acnes* suspension, we measured increases in KC and TNF-α production in the air pouch of male and female mice. This increase was significant for both mediators with BPI infl compared to the control condition (**Figures 5A, B**). We measured immune cell recruitment in response to *C. acnes* injection, which was mainly driven by PMNs, *via* flow cytometry (**Figures 5C, D**). In male mice, the median PMN recruitment was approximately 90% for the three strains compared to 30% for the control group. In female mice, median PMN recruitment was approximately 80% with the *C. acnes* strains compared to 20% in the control condition, and maximum PMN recruitment was reached with the BPI inf strain (median of 94%) (**Figure 5E**). In male mice, all three strains induced a substantial increase in the number of PMNs in the air pouch: approximately 10^7^ cells compared to 2×10^5^ in the control condition. Among the female mice, the highest recruitment (median value of 1.16×10^7^ PMN) was observed in the BPI infl group, in which only 3.64×10^4^ PMNs were recruited (**Figure 5F**). Immunofluorescence staining and Masson’s trichrome staining of histological sections revealed more cells in the ventral and back membranes of the air pouches, including more PMNs; these increases were associated with injection of the *C. acnes* suspension (**Figures 5G–J**).

Notably, PMN recruitment induced by 10^7^ bacteria at 6 h post-injection gradually declined until it was no longer significant after 24 h for both BPI-related clinical strains and after 48 h for all strains. In addition, a lower bacterial concentration (10^6^ bacteria) was not sufficient to induce significant PMN recruitment in the air pouch regardless of infection time, whereas with a higher bacterial dose (10^8^ bacteria), significant neutrophil recruitment was maintained for up to 24 h post-infection for all strains, but there were no differences between the strains (**Supplementary Figure 2**).

### 3.7 PMN response to *C. acnes* in an *in vitro* model mimicking BPI

To determine whether *C. acnes* is detected by neutrophils in an environment mimicking an infected bone site with material, we established a more complex *in vitro* model to decipher whether CIP and BPI inf still behave differently. This tripartite model included osteoblasts, neutrophils, and *C. acnes* strains on titanium alloy disks. Human primary osteoblasts cultured on titanium alloy disks exhibited a spread-out morphology and provided good coverage of the material surface (**Supplementary Figure 3A**). Over 24 h, human primary osteoblasts produced IL-6, IL-8, and MCP-1, with median levels of 165 pg/mL, 32 pg/mL, and 421 pg/mL, respectively. Neither TNF-α nor MIP-1β production was reported over this period (**Supplementary Figure 3B**).

**Figure 6:**
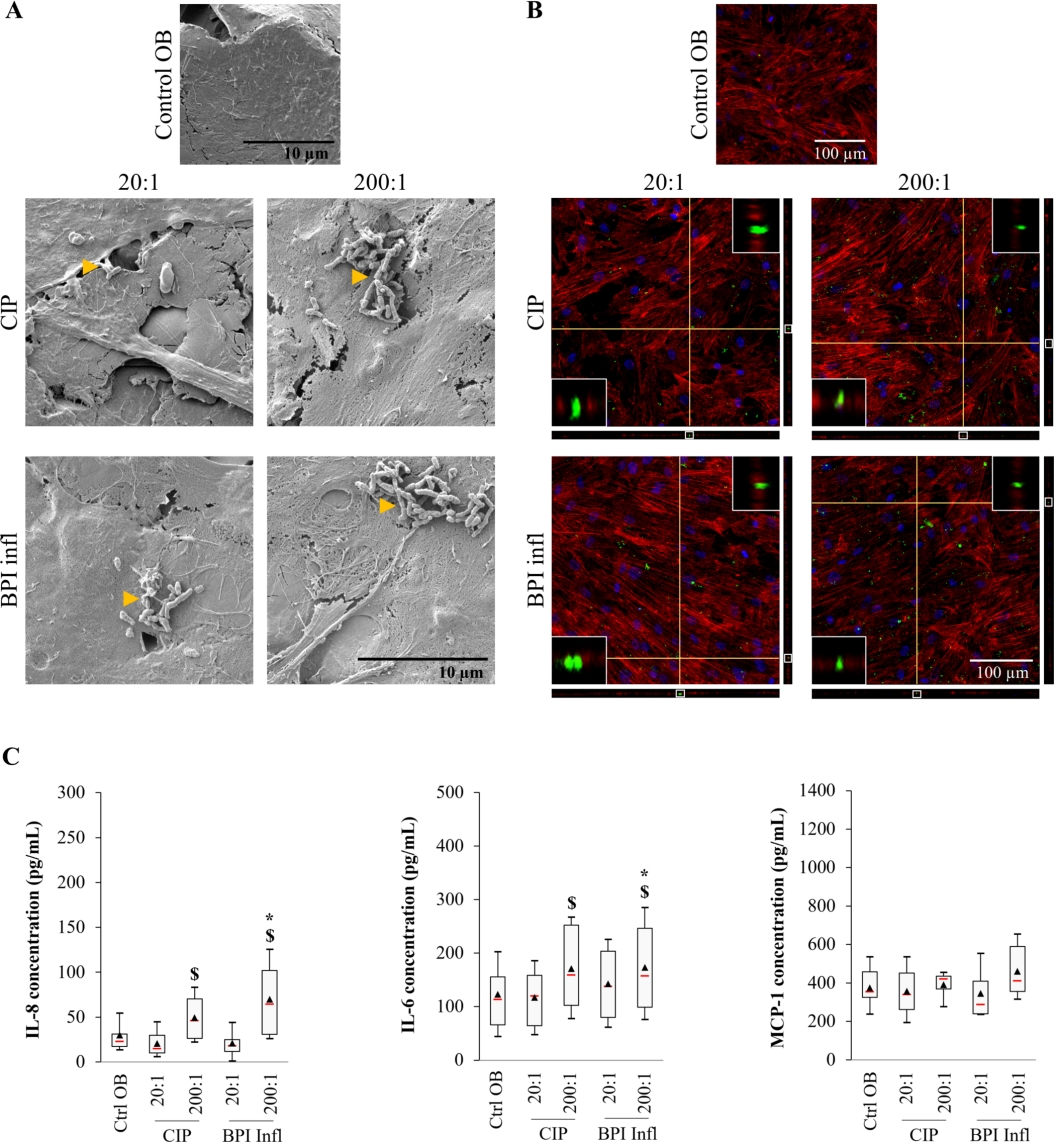
*C*. *acnes* invades osteoblasts and stimulates the production of proinflammatory mediators. **(A)** Representative SEM images (magnification ×5000, yellow triangles indicate bacteria) and **(B)** CLSM images (DAPI-stained nuclei in blue, phalloidin-AlexaFluor^®^568-stained actin filaments in red, AlexaFluor^®^488-immunostained *C*. *acnes* in green) of human primary osteoblasts infected with the CIP or BPI infl *C*. *acnes* strains at two MOIs after 1 h of penicillin/streptomycin treatment and further incubation for 24 h. Each fluorescence image is associated with XZ and YZ projections, and the inserts at the top right and bottom left of each image are enlarged versions of the portion framed in white of the YZ and XZ projections, respectively. **(C)** The secretion of proinflammatory mediators by osteoblasts in response to *C*. *acnes* after 24 h of culture was determined by ELISA. *p<0.05 *vs.* ctrl OB, $*p*<0.05 between MOIs, n=8 independent biological replicates from 4 independent osteoblast donors.

Twenty-four hours post-infection with *C. acnes*, we used SEM to observe bacteria attached to the surface of osteoblasts. The bacteria were more easily visible at an MOI of 200:1 than at an MOI of 20:1 (**Figure 6A**). The antibiotics had not impact after infection, as SEM images obtained without penicillin−streptomycin treatment revealed a similar presence of bacteria at the cell surface at an MOI of 200:1 (**Supplementary Figure 4A**). Confocal laser scanning microscopy (CLSM) also revealed bacteria inside osteoblasts cultured with both *C. acnes* strains, including at the lowest MOI (**Figure 6B**, **Supplementary Figure 4B**). IL-6 and IL-8 secretion was moderately induced by *C. acnes* in an MOI-dependent manner (*p*<0.05 between MOIs). The same trend was observed for BPI infl-induced MCP-1 production (**Figure 6C**). Mediator secretion increased 1.4-fold for IL-6, 2.4-fold for IL-8, and 1.2-fold for MCP-1 in response to *C. acnes* at an MOI of 200:1 in comparison with the uninfected control (median values). Without antibiotic treatment, cytokine production by infected osteoblasts was similar. The secretion of the mediators IL-6, IL-8, and MCP-1 increased 1.8-, 4.5- and 1.5-fold, respectively, in response to *C. acnes* at an MOI of 200:1 compared to that in the uninfected control (median values, **Supplementary Table 4**).

To assess the proinflammatory effect of mediators released by infected osteoblasts on PMNs, the latter were cultured with osteoblast-conditioned supernatants for 4 h. We observed slight morphological changes to PMNs in the presence of conditioned supernatants, particularly those from infected osteoblasts at an MOI of 200:1 (**Supplementary Figure 5A**). The cells exhibited smoother membranes with more and tighter cell−cell contacts. After 4 h of incubation, there was a small amount of IL-8, which varied in a *C. acnes* MOI-dependent manner (*p*<0.05 between MOIs). The median production of IL-6 was 95 pg/mL, with no significant difference between conditions. For MCP-1, only the OB/CIP 200:1 conditioned supernatant appeared superior to the uninfected OB control (**Supplementary Figure 5B**).

**Figure 7:**
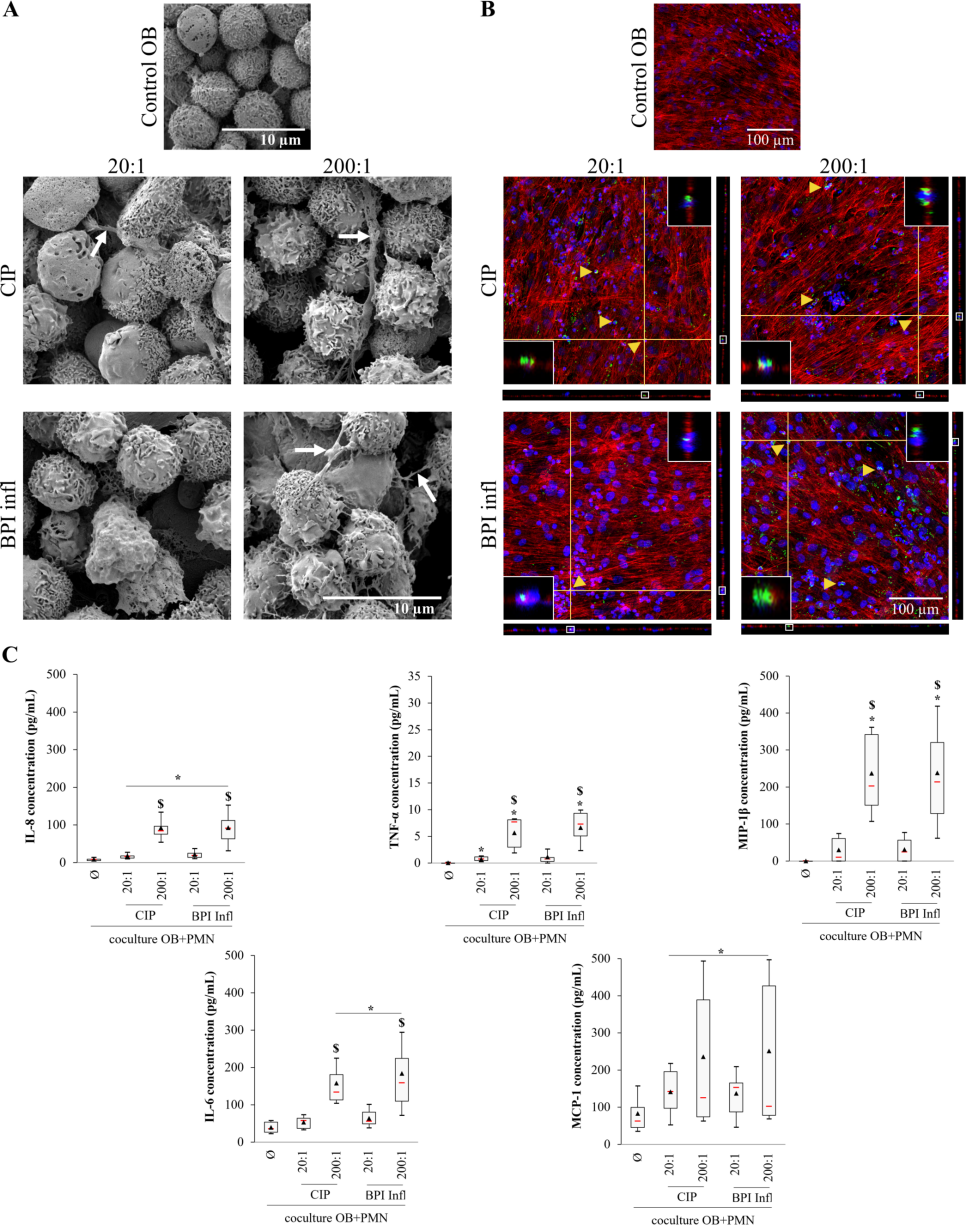
A complex interaction among bone cells, immune cells and *C. acnes* leads to the production of proinflammatory mediators. **(A)** Representative SEM images (magnification ×5000, white arrows indicate NETs) and **(B)** CLSM images (DAPI-stained nuclei in blue, phalloidin-AlexaFluor^®^568-stained actin filaments in red, AlexaFluor^®^488-immunostained *C*. *acnes* in green, yellow triangles indicate bacteria inside PMNs) of primary osteoblasts infected with CIP or BPI infl *C*. *acnes* strains following incubation with PMNs. Each fluorescence image is associated with XZ and YZ projections. The inserts at the top right and bottom left of each image are magnified images of the portion framed in white on the YZ and XZ projections, respectively. **(C)** The secretion of proinflammatory mediators by human primary osteoblasts and/or PMNs induced by *C*. *acnes* was measured. *p<0.05 *vs.* control OB+PMNs, $*p*<0.05 between MOIs, n=8 independent PMN donors on 4 independent osteoblast donors.

After PMN were directly added to infected osteoblasts, SEM images revealed PMN adhesion and there were no visible bacteria. PMNs showed morphological signs of activation, with structures resembling NETs (**Figure 7A**). CLSM revealed the presence of both *C. acnes* strains inside PMNs, indicating that the bacteria probably remained at the osteoblast surface (**Figure 6A**) before being engulfed by PMNs (**Figure 7B**). In terms of proinflammatory mediators, there was significant IL-8 production, with a median level of 90 pg/mL, after the tripartite interaction; IL-8 production occurred in a *C. acnes* MOI-dependent manner, and production increased compared to the control OB/PMN coculture without bacteria (*p*<0.05 *vs*. control; *p*<0.05 between MOIs) (**Figure 7C**). Over the same incubation period but without PMN addition, there was a low IL-8 concentration of approximately 15 pg/mL (median value) (**Supplementary Figure 6**). There were significant increases in TNF-α, MIP-1β, and IL-6 secretion after tripartite interaction with both strains at an MOI of 200:1 compared with that of the control OB/PMN group at an MOI of 20:1 (**Figure 7C**); however, there was no significant TNF-α, MIP-1β, but a slight IL-6 production (approximately 43 pg/mL) in the absence of PMNs (**Supplementary Figure 6**). Finally, MCP-1 production increased in response to *C. acnes* (**Figure 7C**). There was no significant MCP-1 production at the same stage without PMN addition when compared to the uninfected control (**Supplementary Figure 6**).

## 4 Discussion

In this work, we aimed to evaluate the PMN immune response triggered by *C. acnes* strains of distinct origins. We were interested in the CIP laboratory strain from facial acne and the BPI and BPI infl clinical strains associated with an implant infection from shoulder prosthesis and spine instrumentation, respectively. Notably, despite their different clinical origins, all three strains belong to the same IA phylotype. Upon clinical examination, the BPI strain was a non-symptomatic strain, and the patient had a normal leukocyte count and a low percentage of PMNs in the blood, whereas BPI infl was associated with clinical symptoms. Importantly, BPI-related clinical strains are not often associated with obvious clinical signs of inflammation, which is why we included the BPI infl strain in our study. This strain induced a more substantial acute inflammatory response *in vitro*, in line with the symptomatic infection in the patient. The genome analysis of our three strains indicated the presence of four additional genes in BPI infl, this gene sequence is very scarce and was not present in the genomes of the other two strains. Among these four genes, one is annotated as *FtsK*, which is a gene coding for a DNA translocase (29) involved in cell division and recombination in *Escherichia coli* and *Staphylococcus aureus* (30, 31). However, we did not find any work in the literature related to the role of FtsK in *C. acnes*. Another gene is a site-specific recombinase belonging to the phage integrase family. The presence of these two genes could promote gene rearrangements to improve the virulence of this strain. However, the limited evidence is not enough to confirm this hypothesis. The two other genes were annotated as hypothetical proteins, one of which may be involved in DNA−protein covalent cross-linking and DNA replication. We found that only five other *C. acnes* genomes possessed the four consecutive genes present in the BPI infl strain for which we performed sequence typing: *C. acnes* TP-CU38926 (ST2 CC2), *C. acnes* TP-CU42627 (ST2 CC2), *C. acnes* CBS-BPNBT19269 (ST115 CC3) (no article related), *C. acnes* HKGB3 (ST2 CC2) and *C. acnes* HKGB4 (ST1 CC1) (with lower correspondence for HKGB4, no article related). These four genes were not found in other bacterial genera or species. Only *C. acnes* TP-CU426, *C. acnes* HKGB3 and *C. acnes* TP-CU389 were ST2 CC2. The BPI infl strain is ST1 CC1, which suggests that these four strains are not phylogenetically related. Notably, the strains TP-CU389 and TP-CU426 possess the plasmid pTZC1, which is a transferable multidrug resistance plasmid (32, 33). However, this plasmid is not present in the BPI infl strain. Despite the information provided by genome sequencing, these data are insufficient to establish a link between these genes and the difference in clinical signs between patients with the two BPI-related clinical strains. Additional studies should be carried out to determine whether these genes transferred into the CIP strain may increase its inflammatory potential, or if deletion of these genes in the BPI infl strain may temper the PMN response.

In the first *in vitro* model, we demonstrated that PMNs detect *C. acnes* independently of clinical origin and culture state. During biofilm infection on a prosthetic implant, antibiotics and host immune defenses are ineffective (34). In addition, the presence of an implant appears necessary for *C. acnes* survival at the infection site based on embedment in a biofilm, as described in an *in vivo* model by Shiono et al. (35). Our results showed that under our conditions, *C. acnes* biofilms did not avoid PMN recognition *in vitro*, as the latter produce cytokines in response to biofilms. Our data are consistent with those of another study claiming that *C. acnes* biofilm formation is considered a key factor in acne pathogenesis and does not limit the inflammatory response, suggesting that the ability of *C. acnes* biofilms to subvert the host immune response is specific to the context of implant-associated infection (36, 37).

To assess whether the limited immune system response to a BPI could take place prior to biofilm formation on the implant surface, we performed experiments with bacteria in a planktonic state. Compared with that of other gram-positive bacteria, the cell wall of *C. acnes* exhibits unusual characteristics. Indeed, the presence of an atypical peptidoglycan containing a cross-linked region of peptide chains was suspected to confer resistance to the degrading and oxidizing enzymes of the host defense system (38). Nevertheless, we identified individual *C. acnes* or small clusters attached to PMNs by SEM, and we showed that our three strains were internalized by PMNs in an MOI-dependent manner. This finding suggests that *C. acnes* does not have the intrinsic ability to escape cell recognition. Moreover, the PMN/*C. acnes* interaction led to a clear cellular response, as indicated by the increased cytokine production depending on the amount of bacteria. The cellular response occurred at the transcription and protein levels and appeared to be greater in the presence of BPI-related clinical strains. The release of the chemokines IL-8 and MIP-1β can attract new PMNs and monocytes to the site (39, 40), while TNF-α neo-synthesis could promote immune cell activation, especially in the presence of the BPI infl strain. Considering that the level of TNF-α increases only with the BPI infl strain, we hypothesize that specific virulence factors for this strain have specific abilities based on genetic differences; this hypothesis aligns with the peculiar profile of the BPI infl patient. This hypothesis will have to be investigated in future studies. Finally, the secretion of MMP-9 in the tertiary granules of PMNs stimulates matrix degradation, thereby facilitating the transendothelial migration of other inflammatory cells (41). Our data suggest that the MMP-9 production observed in response to BPI infl is mainly due to PMN degranulation, as we did not observe any variation in *MMP9* gene expression. The absence of LDH signal variation confirmed that the mediators found in culture supernatants are the consequence of immune cell activation and not only cell lysis. Taken together, these results show that PMNs could initiate an effective acute inflammatory response. IL-6 and MCP-1 have also attracted our interest as their production has been reported in *C. acnes* infections in degenerated intervertebral discs. MCP-1 is a chemokine produced by many cell types, including PMNs in some peculiar clinical contexts, and is known for its recruitment of monocytes (42, 43). IL-6 secretion by PMNs *in vitro* is debated. On the one hand, it has been reported that human PMNs can produce IL-6 with autocrine TNF-α (44), and on the other hand, IL-6 secretion was ultimately attributed to the small percentage of contaminating monocytes in *in vitro* culture (45). Our results did not show significant production of these two mediators following direct interactions with our *C. acnes* strains.

To further investigate the secretion of proinflammatory mediators, we focused on degranulation, a mechanism that allows PMNs to release the content of their intracellular granules into the environment. Mature PMNs have a cytoplasm that is enriched with four types of granules that store various antimicrobial substances (18, 20, 46). During the activation process, secretory granules (containing cytokines such as IL-8) are the first to be degranulated. This degranulation is followed by the release of tertiary granules (containing MMP-9, arginase-1, and lysozyme), leading to an increase in surface expression of the CD16 receptor. Exocytosis of secondary granules (storing cathelicidin, lactoferrin and lipocalin 2) leads to an increased level of the CD66b receptor. The degranulation process is ultimately completed by the release of primary granules (containing defensins, myeloperoxidase and neutrophil elastase), resulting in an increase in the surface expression of the CD63 receptor and shedding of the CD16 receptor, resulting from cleavage by PMN proteases and/or membrane reuptake (21, 22). BPI infl induced more variation in surface receptor expression in favor of the degranulation process than did the other strains; this finding is consistent with the results of the release of proinflammatory mediators. Surprisingly, despite the full activation of PMNs accompanied by the release of highly antimicrobial substances (47), we never found substantial alterations to bacterial structure by SEM, in biofilms or planktonic bacteria. Further analyses are needed to determine whether the PMN antibacterial response is inhibited by *C. acnes* or whether the corresponding targets in *C. acnes* remain accessible.

As PMNs could detect all *C. acnes* strains, despite differences in response, we investigated whether nonimmune cells from connective tissues could detect *C. acnes* and then recruit immune cells to trigger the inflammatory response. In a murine air pouch model, which enabled us to obtain an environment closely resembling a synovial cavity (48, 49), we demonstrated PMN recruitment to the air pouch 6 h after the injection of bacterial suspension with an increased trend with the BPI infl strain in female mice. Resident cells from the air pouch membrane could detect *C. acnes* and recruit PMNs from the bloodstream toward the infection site. This finding invalidates the hypothesis of a reduced *C. acnes*-triggered inflammatory response in patients due to defective recognition by nonimmune resident cells near the implanted material. In addition, importantly, in this model, male mice exhibited a more robust response than female mice (up to 20% more leukocytes recruited); this result poses new hypotheses related to gender imbalance in patients, aside from an increase in the number of pilosebaceous glands (50). This gender imbalance is supported by studies showing that females are more prone to be responsive in this air pouch model (51).

Intracellular invasion by a variety of pathogenic species allows them to escape the host immune system and survive inside the body. For example, the ability of *S. aureus* to be internalized inside osteoblasts is a key strategy that the bacterium uses to protect itself and maintain infection at the bone site (52). However, osteoblasts respond to *S. aureus* internalization by secreting inflammatory factors, which, in turn, activate and recruit immune cells (53). Finally, we studied whether *C. acnes* was detected in the same way by PMNs in an *in vitro* model using human primary osteoblasts, human primary PMNs, and *C. acnes* strains on titanium alloy discs to mimic an infected bone site with prosthetic material. Our results showed that the mediators secreted by infected osteoblasts did not activate PMNs, as the levels of mediators following the indirect interaction were similar to those initially provided in the conditioned supernatants (one-half dilution of conditioned supernatant in PMN culture medium). Following the tripartite interaction, IL-8, TNF-α, and MIP-1β production was mainly mediated by PMNs. In fact, infected osteoblasts without PMNs produced no TNF-α or MIP-1β and only a small amount of IL-8 over the same period. Thus, cell−cell contact is required to elicit the production of these three proinflammatory mediators by PMNs, as conditioned supernatants does not trigger this process. We have shown that PMNs do not secrete IL-6 or MCP-1 following direct interactions with *C. acnes*. Consequently, the production of these two mediators after tripartite interaction results from secretion by osteoblasts. However, IL-6 and MCP-1 production by infected osteoblasts without PMNs was much lower than that observed after OB/PMNs/*C. acnes* interactions. Considering these results, we assume that IL-6 and MCP-1 production by osteoblasts is dependent on both neutrophils and bacteria.

Our tripartite interaction model suggested that *C. acnes* may escape the immune system through at least partial internalization into osteoblasts, a phenomenon expected to reduce the bacterial load available to PMNs. Considering the MOI-dependent response of PMNs in this study, there may have been moderation of the immune response triggered by *C. acnes* in patients. The ability of *C. acnes* to behave as an intracellular pathogen in different cell types, including human bone cells, has been described (54–56). Previous work in our laboratory has shown that internalized *C. acnes* persist intracellularly for up to 48 h within osteoblast cells (14). The long-term fate of bacteria at bone sites has not yet been well studied, and this topic should be studied more closely, as it has been shown that the pathogenic potential of *C. acnes* truly depends on the anatomical site of infection (55). While a difference in the response to different strains was highlighted in the first *in vitro* model, in the tripartite model, the BPI infl strain did not induce a greater inflammatory response than CIP strain.

In conclusion, this work provided evidence that despite the different clinical origins, *C. acnes* strains can be detected by PMNs, the first line of defense of the immune system, both in planktonic culture and biofilm state. Bacteria triggered PMN activation and degranulation in a dose-dependent manner, with a more sustained inflammatory response in the presence of a clinical strain that induced clinical signs of inflammation in the patient. Among the major strengths of our work, the use of human primary cells may provide insights into the translational relevance of our findings. Our data further demonstrates the importance of thinking about cellular protagonists as a whole to understand the response of osteoblasts and immune cells to *C. acnes*-related BPIs. With osteoblasts, PMNs were still able to detect *C. acnes* strains, however with no longer difference according to their clinical inflammatory profile or not. Although the precise mechanism underlying the lack of clinical signs in most *C. acnes*-related BPIs has not been elucidated, osteoblasts appear to temper the immune response to *C. acnes*.

## Data Availability

The original contributions presented in the study are included in the article/**Supplementary Material**, further inquiries can be directed to the corresponding author/s. Raw reads for the strains sequenced in this study (CIP, BPI, and BPI infl) are available on European Nucleotide Archive (ENA) website under the project name PRJEB82770.
