# Supplementary material for: Cutibacterium acnes strains associated with bone prosthesis infections cannot evade the host immune system
Source: Front Immunol. 2024 Nov 27;15:1468709. doi: 10.3389/fimmu.2024.1468709 (PMC11632127; doi:10.3389/fimmu.2024.1468709)

Supplementary Material

#
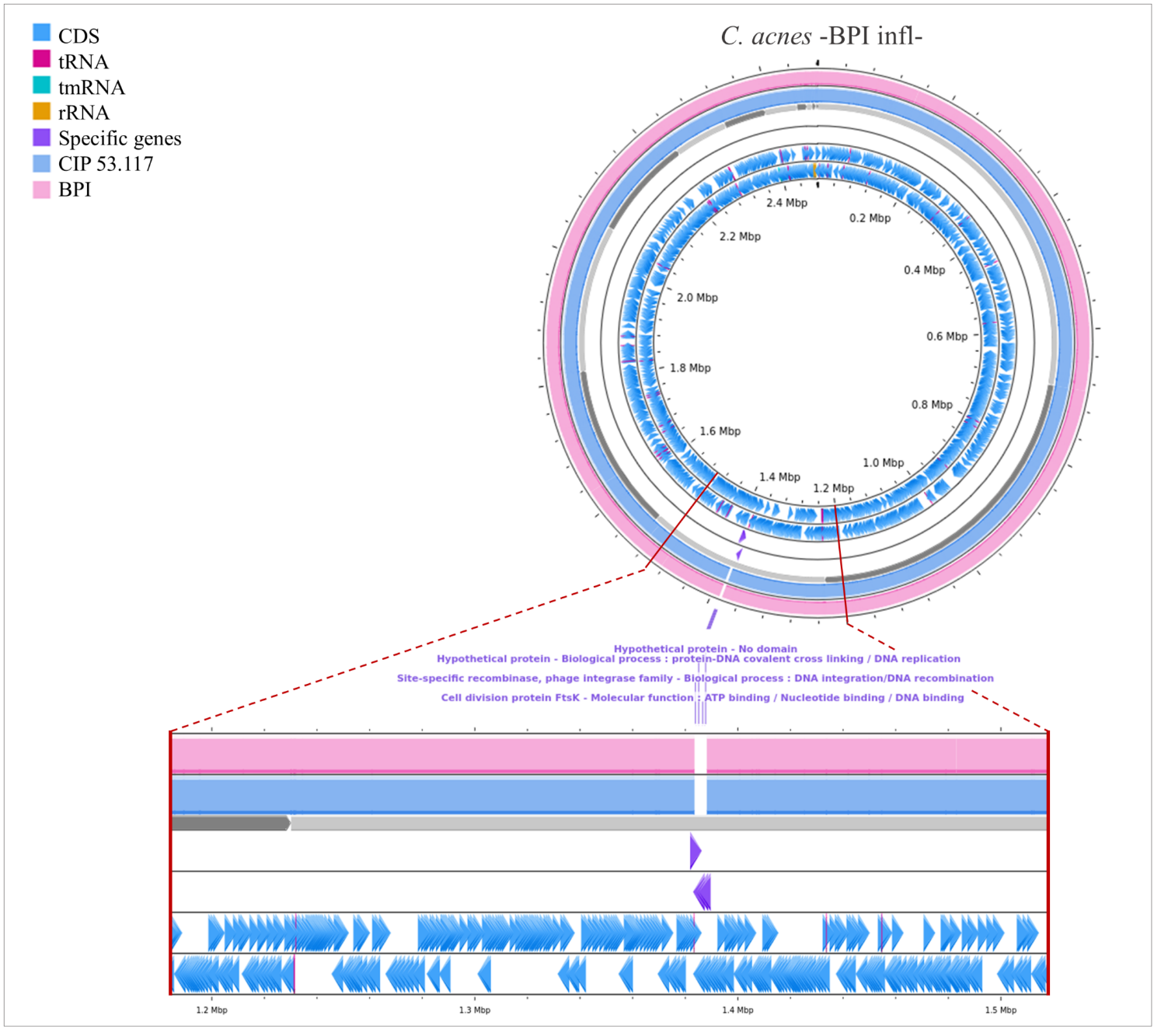
Supplementary Figures

**Supplementary Figure 1:** **The BPI infl strain possesses four consecutive additional genes.** Whole-genome sequencing of the three *C. acnes* strains was performed using Illumina short-read technology and revealed that the BPI infl strain has four consecutive genes that are present in its genome but not in the CIP and BPI genomes.


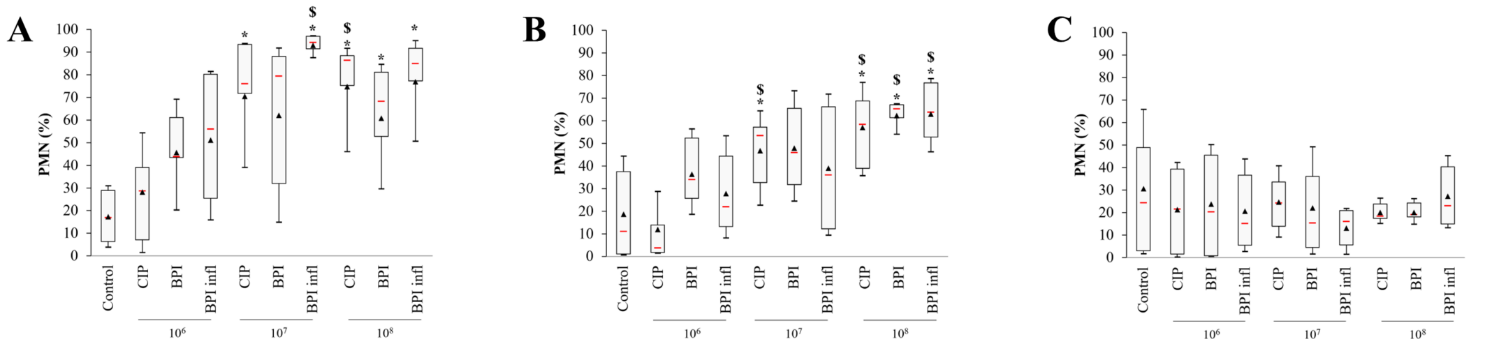


**Supplementary Figure 2:** **Dose- and time-dependent effects of *C. acnes* on PMN recruitment.** Quantification of neutrophils recruited into the air pouch of female mice **(A)** 6 h, **(B)** 24 h or **(C)** 48 h after injection of PBS (control) or a *C. acnes* suspension with 10^6^,10^7^ or 10^8^ bacteria, was performed by flow cytometry. * *p*<0.05 *vs.* control, $ *p*<0.05 *vs.* 10^6^, n=6 mice per group.


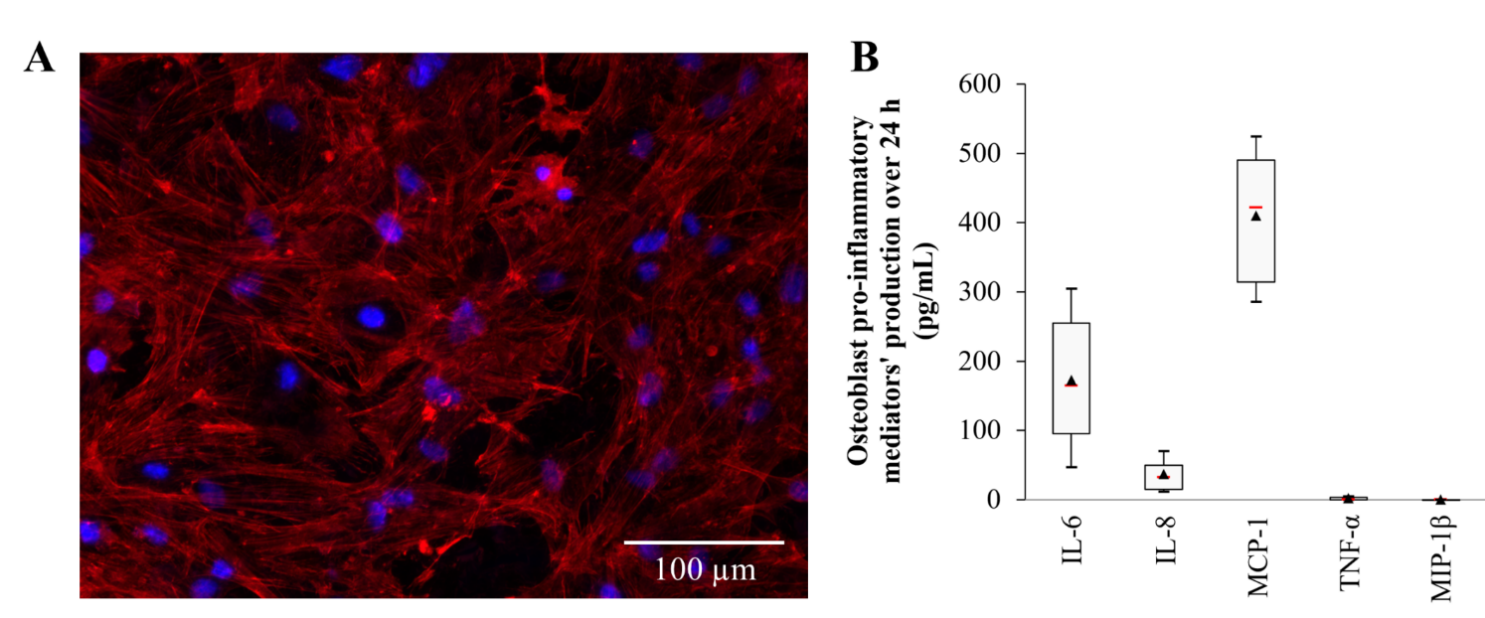


**Supplementary Figure 3:** **Human primary osteoblasts readily colonize titanium and secrete proinflammatory mediators. (A)** Representative fluorescence staining of primary osteoblasts cultured on titanium alloy for 4 days (DAPI-stained nuclei in blue, Phalloidin-AlexaFluor^®^568-stained actin filaments in red). ×20 objective. Scale bar = 100 µm. **(B)** The production of proinflammatory mediators by osteoblasts over a 24 h period was measured by ELISA in cell culture supernatants. n=8 independent biological replicates from 4 independent donors.


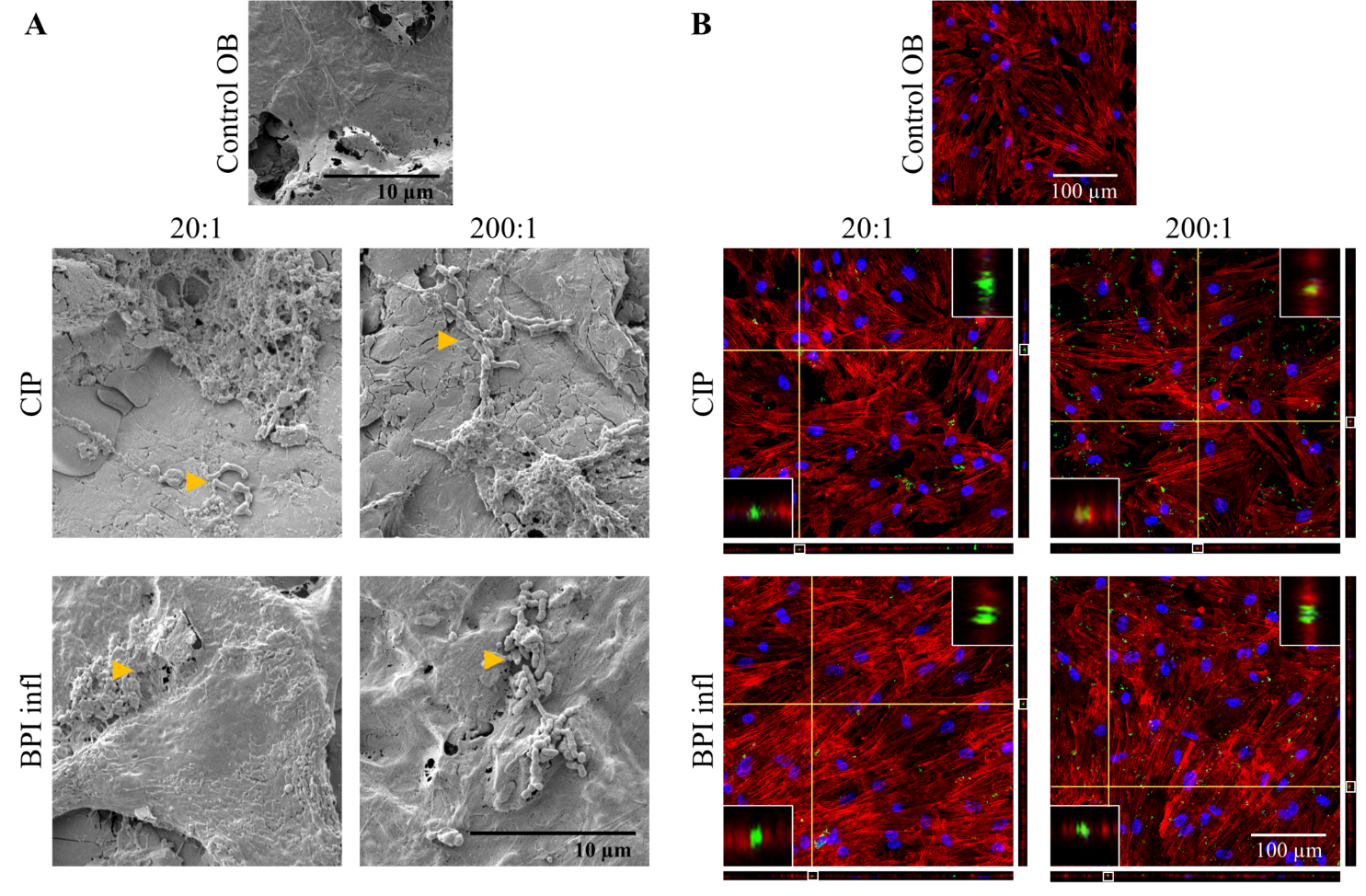


**Supplementary Figure 4:** **Antibiotic treatment does not impact *C. acnes* osteoblast invasion.** **(A)** Representative SEM images (magnification ×5000, yellow triangles indicate bacteria) and **(B)** CLSM images (DAPI-stained nuclei in blue, phalloidin-AlexaFluor^®^568-stained actin filaments in red, AlexaFluor^®^488-immunostained *C. acnes* in green) of human primary osteoblasts infected with the CIP and BPI infl *C. acnes* strains at two MOIs without antibiotic treatment and after an additional 24 h of incubation. Each fluorescence image is associated with XZ and YZ projections, and the inserts at the top right and bottom left of each image are enlarged images of the portions framed in white on the YZ and XZ projections, respectively.


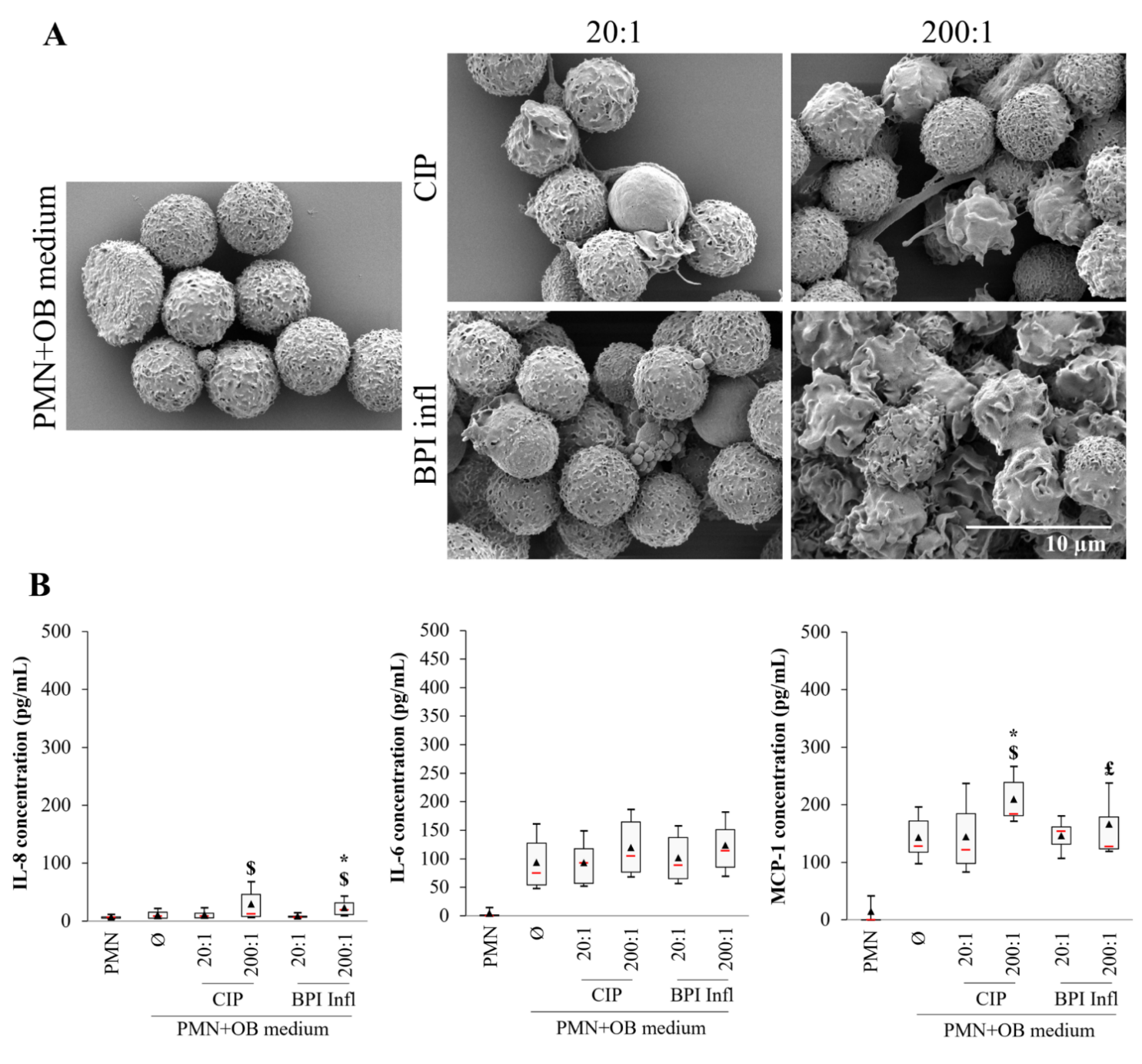


**Supplementary Figure 5: Osteoblasts conditioned supernatants do not activate PMNs.** **(A)** Representative (n=4 osteoblast and PMN independent donors) SEM images (magnification ×5000) of human primary PMNs cultured in the conditioned supernatants of infected human primary osteoblasts. **(B)** Concentration of proinflammatory mediators in PMN supernatants cultured in conditioned medium from infected human primary osteoblasts. * *p*<0.05 *vs.* control OB, $ *p*<0.05 between MOIs, £ *p*<0.05 *vs.* CIP, n=8 independent PMN donors on 4 independent osteoblast donors.


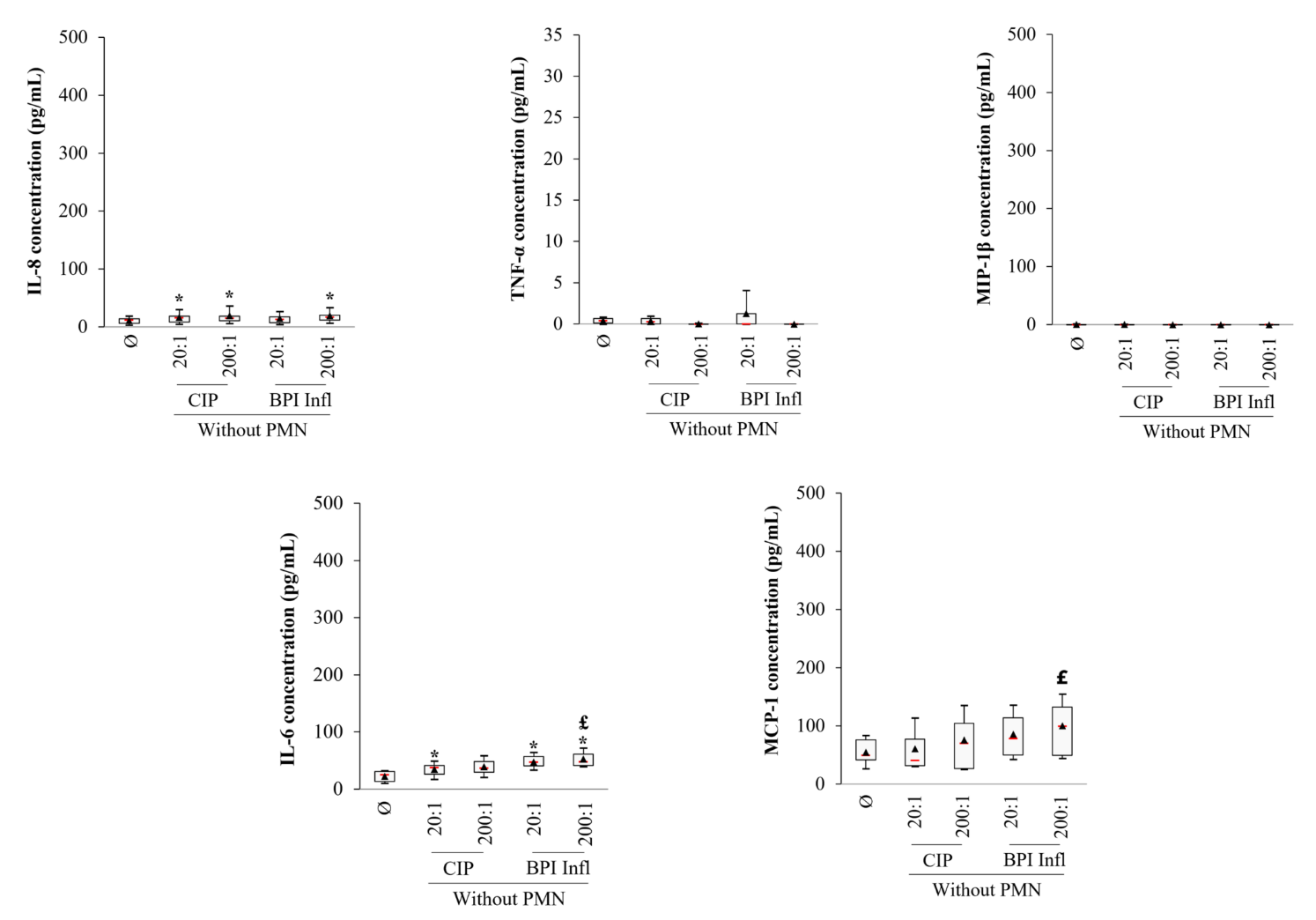


**Supplementary Figure 6: Slight production of proinflammatory mediators by osteoblasts without PMN addition.** Changes in the secretion of proinflammatory mediators by osteoblasts in response to incubation with *C. acnes* for 4 h without PMN addition (ELISA). * *p*<0.05 *vs.* control OB, £ *p*<0.05 *vs.* CIP, n=8 independent osteoblast donors.

# Supplementary Tables


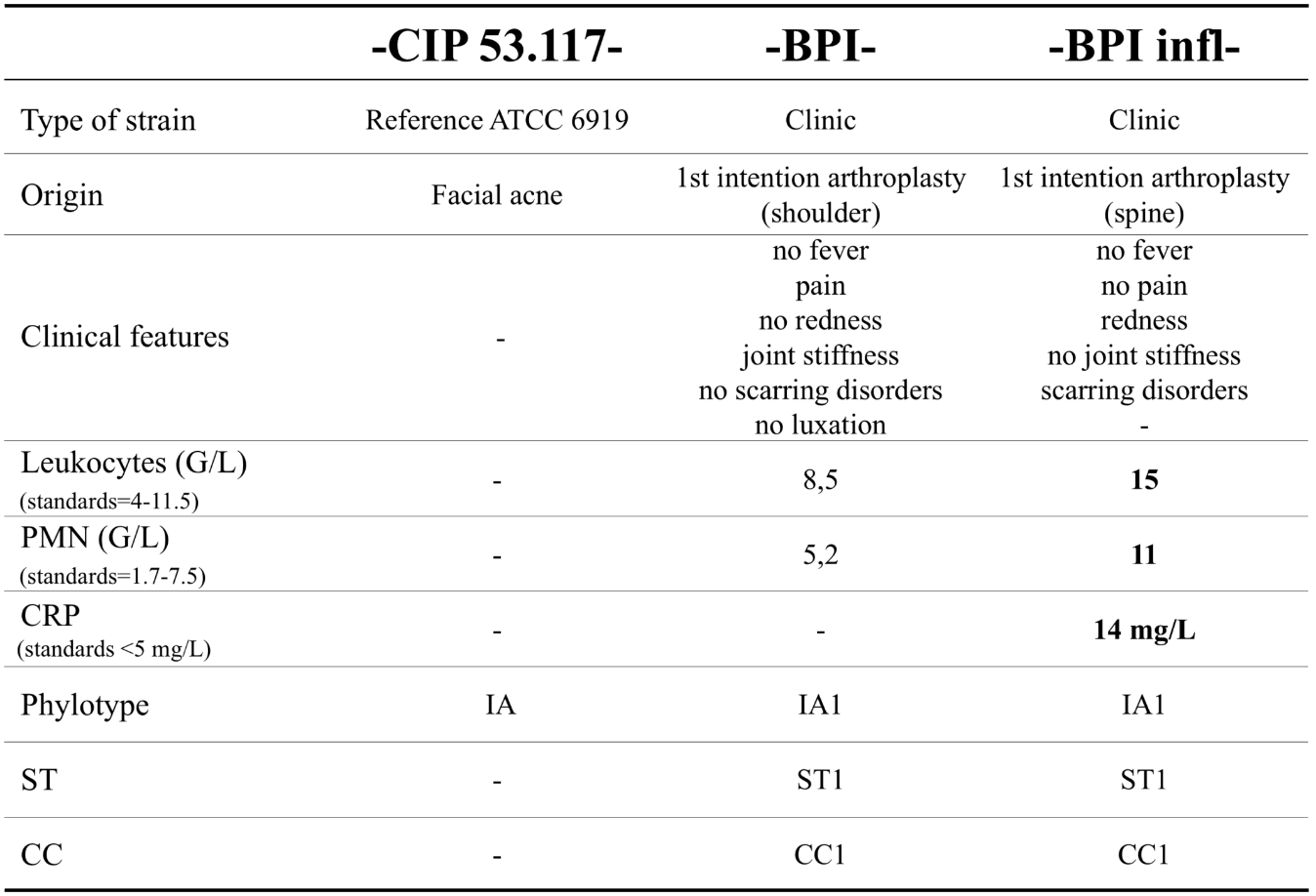
**Supplementary Table I:** **Clinical features of the *C. acnes* strains studied.**


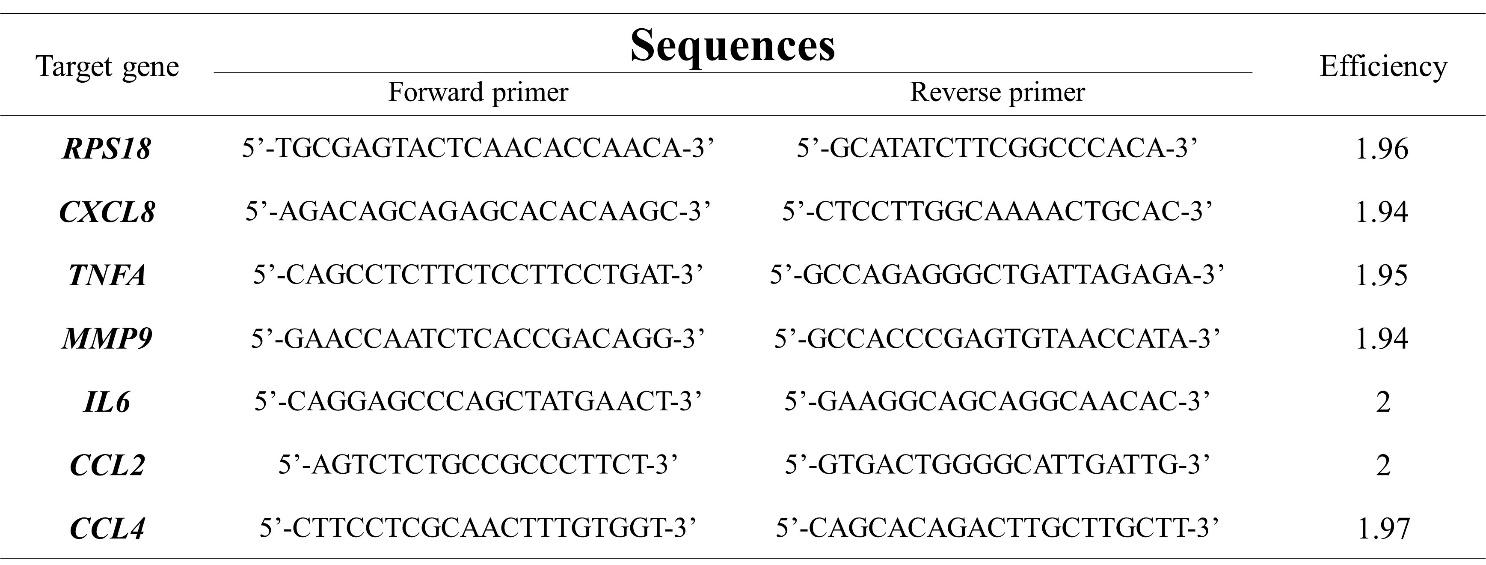
**Supplementary Table II: Primer nucleotide sequences used for RT‒qPCR and primer efficiency for each pair of primers.**

**
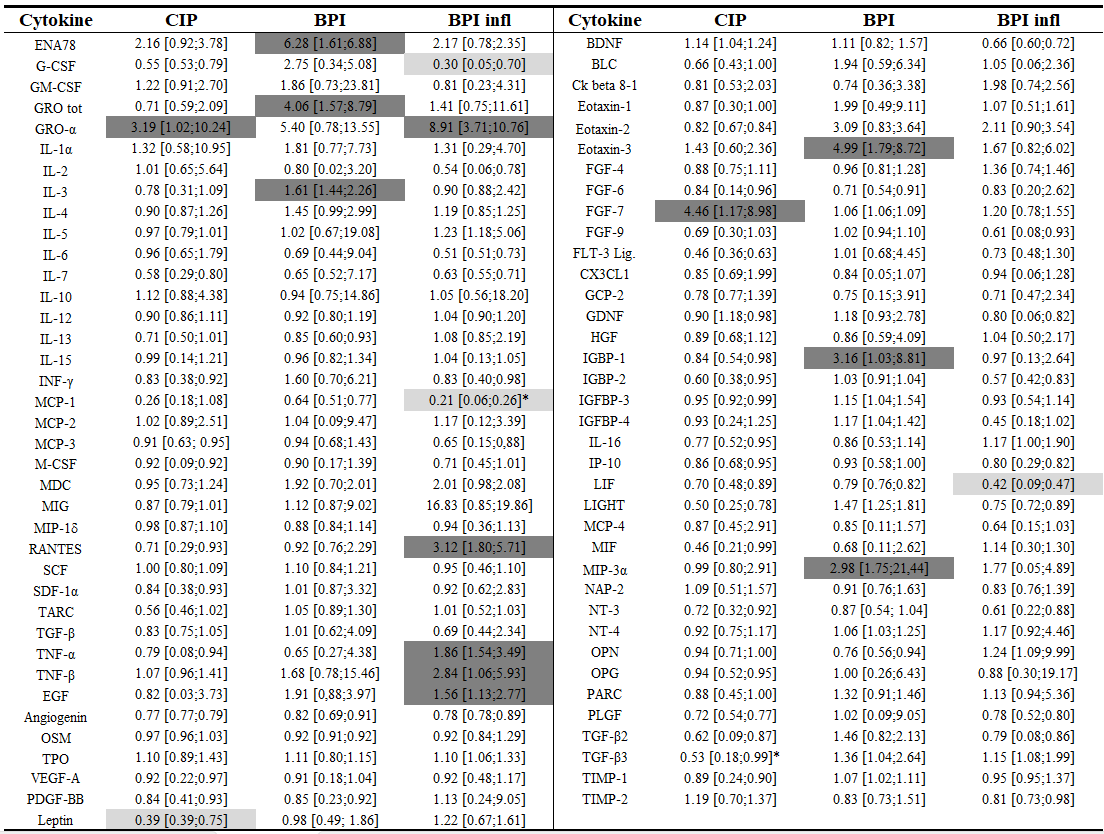
Supplementary Table III:** **Effect of planktonic *C. acnes* strains on PMN cytokine production.** Cytokine production of the control and PMNs following interaction with planktonic *C. acnes* strains was measured by an antibody array, and the ratio is here reported. The data are presented as the median [1^st^ quartile; 3^rd^ quartile] for each cytokine evaluated. For at least 4 of the 5 donors, the variations highlighted in dark gray indicate an increase of at least 1.49-fold compared with the control condition, and values under 0.43 (decrease *vs.* control threshold) compared with the control condition are highlighted in light gray.

**Supplementary Table IV:** ***C. acnes* triggers the secretion of a proinflammatory mediator by *C. acnes*-infected osteoblasts, conditioned supernatants, or infected osteoblasts following incubation with PMNs, all without antibiotic treatment, after 24 h of culture** (ELISA). The data are presented as the 1^st^ quartile, median and 3^rd^ quartile for each cytokine evaluated. Bold values *p*<0.05 *vs.* control OB or control OB-conditioned supernatant, $ *p*<0.05 between MOI. n=8 independent biological replicates from 4 independent donors.


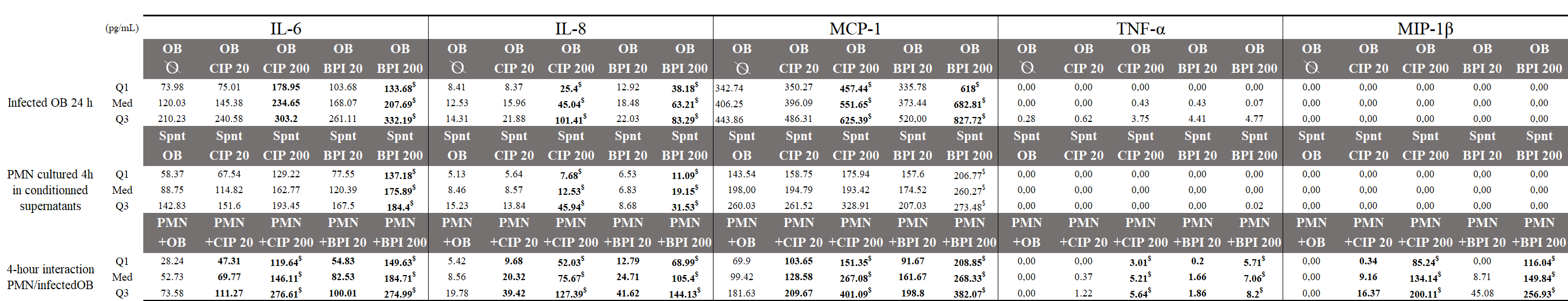

Supplement: Supplementary file 1 [file Table1.docx]
